# Two distinct classes of QTL determine rust resistance in sorghum

**DOI:** 10.1186/s12870-014-0366-4

**Published:** 2014-12-31

**Authors:** Xuemin Wang, Emma Mace, Colleen Hunt, Alan Cruickshank, Robert Henzell, Heidi Parkes, David Jordan

**Affiliations:** Queensland Alliance for Agriculture and Food Innovation, The University of Queensland, Warwick, QLD Australia; Department of Agriculture, Fisheries & Forestry (DAFF), Warwick, QLD Australia; Department of Agriculture, Fisheries & Forestry (DAFF), Stanthorpe, QLD Australia

**Keywords:** Rust resistance, Sorghum, Pleiotropy, Height, Maturity, Stay-green, QTL mapping, Association mapping

## Abstract

**Background:**

Agriculture is facing enormous challenges to feed a growing population in the face of rapidly evolving pests and pathogens. The rusts, in particular, are a major pathogen of cereal crops with the potential to cause large reductions in yield. Improving stable disease resistance is an on-going major and challenging focus for many plant breeding programs, due to the rapidly evolving nature of the pathogen. Sorghum is a major summer cereal crop that is also a host for a rust pathogen *Puccinia purpurea,* which occurs in almost all sorghum growing areas of the world, causing direct and indirect yield losses in sorghum worldwide, however knowledge about its genetic control is still limited. In order to further investigate this issue, QTL and association mapping methods were implemented to study rust resistance in three bi-parental populations and an association mapping set of elite breeding lines in different environments.

**Results:**

In total, 64 significant or highly significant QTL and 21 suggestive rust resistance QTL were identified representing 55 unique genomic regions. Comparisons across populations within the current study and with rust QTL identified previously in both sorghum and maize revealed a high degree of correspondence in QTL location. Negative phenotypic correlations were observed between rust, maturity and height, indicating a trend for both early maturing and shorter genotypes to be more susceptible to rust.

**Conclusions:**

The significant amount of QTL co-location across traits, in addition to the consistency in the direction of QTL allele effects, has provided evidence to support pleiotropic QTL action across rust, height, maturity and stay-green, supporting the role of carbon stress in susceptibility to rust. Classical rust resistance QTL regions that did not co-locate with height, maturity or stay-green QTL were found to be significantly enriched for the defence-related NBS-encoding gene family, in contrast to the lack of defence-related gene enrichment in multi-trait effect rust resistance QTL. The distinction of disease resistance QTL hot-spots, enriched with defence-related gene families from QTL which impact on development and partitioning, provides plant breeders with knowledge which will allow for fast-tracking varieties with both durable pathogen resistance and appropriate adaptive traits.

**Electronic supplementary material:**

The online version of this article (doi:10.1186/s12870-014-0366-4) contains supplementary material, which is available to authorized users.

## Background

Agriculture is facing enormous challenges to feed a growing population in the face of rapidly evolving pests and pathogens. A critical component for addressing these challenges is to breed for increased disease resistance in crop species to avoid the need for costly and potentially environmentally damaging pesticides. The major cereal crops feed over two thirds of the world’s population and yet the production of these crops continues to be challenged by pests and diseases with at least 30% of global food production lost to pathogens [1,2]. Sorghum (*Sorghum bicolor* (L.) Moench) is a C4 cereal grain crop that provides staple food for over 500 million people in the semi-arid tropics of Africa and Asia, in addition to being an important source of feed for livestock. Amongst the cereals, sorghum is one of the best adapted to drought and high temperatures, and will play an increasingly important role in meeting the challenges of feeding the world’s growing population. In recent times, sorghum has become an attractive feedstock alternative for use in the production of bio-ethanol fuel. However, its productivity is often jeopardised by foliar fungal diseases. Among the fungal diseases, leaf rust causes significant crop damage in sorghum [3], with rust diseases being among the most widespread and economically important diseases of cereals world-wide, e.g. [4].

Sorghum rust, caused by *Puccinia purpurea* Cooke, is widely distributed and occurs in almost all sorghum growing areas of the world [5]. Leaf rust frequently predisposes sorghum to other major diseases and agronomic problems, such as the Fusarium stalk rots, charcoal rot, grain mould, anthracnose and lodging [6-8]. In addition to indirect yield losses through predisposition to other diseases, sorghum leaf rust can cause direct yield losses of up to 50% depending on favourable environmental conditions for disease development and cultivar susceptibility [9]. In Australia, leaf rust has the most significant impact on grain yield of all fungal leaf diseases, causing up to 13% yield losses [10]. The disease is seen every year on most sorghum crops, usually after flowering, when the leaves may be covered by the uredinia and telia of the fungus [6]. Due to the influence of environmental and climatic conditions on the development of the disease and the cost of fungicides, genetic resistance offers the best long-term solution for the management of leaf rust in sorghum. As such, better understanding of the genetic control of leaf rust resistance would provide sorghum breeders with critical knowledge to develop new resistant varieties.

The genetic architecture of complex traits is frequently controlled by multiple genes or alleles that vary with genetic background [11]. A single mapping population study can therefore explain only a small part of the global genetic architecture of a complex trait and limits the identification of potential genomic regions due to the absence or monomorphic presence of alleles contributing to the genetic control of a complex trait such as leaf rust resistance [12]. Initial studies into the inheritance of leaf rust resistance in sorghum identified a single dominant gene, *Pu*, conferring rust resistance in sweet sorghum crosses [13]. However, subsequent studies identified rust resistance to be polygenic in nature, with multiple genes and genomic regions identified [3,5,7,14,15]. Therefore, the comparison of QTL detected in multiple populations and multiple environments is particularly important for dissecting the genetic control of polygenic disease resistance and permits investigation of the degree to which the underlying genes contribute to variation in the phenotype under different genetic backgrounds and environmental conditions [16]. Furthermore, traits associated with the physiological development and age of the plant have been implicated in indirectly impacting disease response, with previous studies demonstrating that growth stage can impact on the degree of disease susceptibility in a range of crops e.g. [17-19]. Therefore, a multi-trait analysis approach enables the investigation of potentially pleiotropic disease response QTL.

In this study, a combination of conventional QTL analysis and genome wide association analysis (GWAS) was used to determine the genetic architecture of leaf rust resistance in sorghum using three bi-parental mapping populations and a set of elite breeding lines phenotyped in hybrid combination with multiple testers. All populations were phenotyped not only for rust infection response, but also for height and maturity. Further, the availability of the sorghum whole genome sequence [20] and genetic linkage map-based resources, e.g. [21,22], provided opportunities to compare QTL for rust resistance identified in the current study with previously reported QTL in both sorghum and maize, in addition to previously reported gene families associated with disease resistance.

## Results

### Phenotypic data variability

The predicted means, ranges, and standard deviations for the traits measured for the progeny of the two RIL populations are detailed in Table 1. Rust infection responses of the five parents in the three bi-parental populations are presented in Additional file 1: Figure S1. Heritability for rust resistance was high in all three bi-parental populations (79.4% in S2; 77.3% in S4; 58.6% in S7). In the S2 population, the female parent (ICSV745) had higher levels of rust resistance than the male parent (R890562); 3.5 versus 8. In the S4 population, the male parent (R931945-2-2) exhibited partial resistance to rust (4), in contrast to the high level of susceptibility (7.75) exhibited by the female parent (IS8525). For S2, the rust resistant parent ICSV745 was much later maturing (84.5 days to flowering versus 66) and much taller (192 cm versus 85 cm) than R890562. Between trait correlation was low (Additional file 2: Table S1). For S4, R931945-2-2 was slightly later maturing and shorter than IS8525. The RILs showed transgressive segregation for all three traits. The predicted means, ranges, and standard deviations for all three traits scored in the S7 population are detailed in Table 2. The *S. bicolor* subsp. *verticilliflorum* parent could not be grown in field trials because of its weedy nature. The predicted means, ranges, and standard deviations for the AYT association mapping set across the 3 male testers and 2 location combinations are detailed in Additional file 2: Table S2. Heritability for rust resistance was found to be higher at the Liverpool Plains site (69.7%), in comparison to the Dalby site (38.6%). The relative rust pressure was found to be reduced at the Dalby site, in comparison to the other trials included in the study, through the comparison of rust response scores of check genotypes (Additional file 2: Table S3). The trials were not highly correlated (Additional file 2: Table S1); genotype × environment interaction was observed using a second order factor analytic (FA) site structure where only 68% of the variation was accounted for by the first factor (Additional file 1: Figure S2), and additionally correlations across sites and within each male tester genotypes were higher than on a per site basis (average R^2^ of 0.68 across the 3 male testers versus 0.62 across the 2 environments).

**Table 1:** **Predicted mean (** $\overline{\boldsymbol{X}}$ **) values of rust infection score, height and maturity for the two RIL populations (S2 and S4) and parental lines in the HRS10 trial, plus the standard deviation (σ) and range (minimum and maximum) for each population**

**Population**	**Trait**	${\overline{\mathbf{X}}}_{\mathbf{parentA}}$	${\overline{\mathbf{X}}}_{\mathbf{parentB}}$	${\overline{\mathbf{X}}}_{\mathbf{progeny}}$	***σ***_**progeny**_	**Min**_**progeny**_	**Max**_**progeny**_
S2	Rust	3.50	8.00	7.10	0.97	4.48	8.85
S4	Rust	7.75	4.00	6.99	1.01	3.99	8.70
S2	DTF	84.50	66.00	62.16	3.15	54.42	74.08
S4	DTF	60.25	62.25	58.76	2.54	53.23	66.29
S2	HGT	192.25	85	98.72	12.73	77.26	143.03
S4	HGT	166	94.75	123.62	16.37	87.70	163.04

**Table 2:** **Predicted mean (** $\overline{\boldsymbol{X}}$ **) values of rust infection score, height and maturity for the BC**_**1**_ **F**_**4**_ **population (S7) across multiple trials, plus the standard deviation (σ) and range (minimum and maximum)**

**Trial**	**Trait**	${\overline{\mathbf{X}}}_{\mathbf{progeny}}$	**σ**_**progeny**_	**Min**_**progeny**_	**Max**_**progeny**_
BIL2003^IRR^	Rust	5.85	0.39	4.80	6.96
BIL2003^nonIRR^	Rust	6.20	0.37	5.16	7.18
BIL2003^overall^	Rust	6.02	0.38	5.01	7.07
BIL2003^IRR^	DTF	56.11	0.89	53.54	59.86
BIL2003^nonIRR^	DTF	57.32	0.70	55.21	60.24
BIL2003^overall^	DTF	60.21	1.28	56.73	66.04
BIL2003^IRR^	HGT	120.34	6.39	95.74	146.04
BIL2003^nonIRR^	HGT	118.33	6.97	93.10	151.14
BIL2003^overall^	HGT	115.63	4.08	98.06	127.41

### QTL analysis

The results of the QTL analysis for each trait in each population are shown in Tables 3, 4, and 5 (Additional file 2: Tables S4-S7; Additional file 1: Figures S3-S5).

**Table 3:** **Summary of rust resistance (QRustR), maturity (QDTF) and height (QHGT) QTL identified in the S2 population, detailing the QTL position, 2-LOD confidence interval (CI), flanking markers, peak LOD value, total trait variance explained (R** ^**2**^ **), additive effect, and significance level**

**QTL ID**	**LG**	**Peak cM** ^**a**^	**CI (cM)**	**Flanking markers**	**LOD**	**R** ^**2**^ ^**b**^	**Additive** ^**c**^	**Sig** ^**d**^
QRustR_S2_2.1	SBI-02	93.81	91.5-106.2	Str66/SG38	2.32	5.97	0.316	*
QRustR_S2_5.1	SBI-05	73.01	45.81-73.01	txs387c/sPb-5892	5.18	15.57	−0.502	***
QRustR_S2_6.1	SBI-06-I	6.41	4.41-6.41	MT2/cdo456	1.97	5.59	0.336	*
QRustR_S2_8.1	SBI-08-I	0	0-8.91	sPb-9299/RG8167	2.84	8.13	−0.367	*
QRustR_S2_8.2	SBI-08-II	3.71	0-18.4	sPb-7823/sPb-1291	6.11	18.47	0.568	***
QDTF_S2_3.1	SBI-03	20.8	13-28.4	SSCIR78/sPb-2309	1.87	8.26	0.921	*
QDTF_S2_3.2	SBI-03	211	210.2-217	ST329r/ST1740	2.62	7.41	0.912	*
QDTF_S2_4.1	SBI-04	2.9	0-12.32	ST1163-1/sPb-9468	2.35	7.84	0.936	*
QDTF_S2_5.1	SBI-05	68	64.6-77.73	txs387c/sPb-5892	2.99	10.43	1.031	*
QDTF_S2_10.1	SBI-10	76.5	75.2-88.73	txs558/GE37	1.97	6.17	0.815	*
QHGT_S2_3.1	SBI-03	107.1	97.6-114.4	ST458/sPb-8349	2.92	6.80	3.585	*
QHGT_S2_6.1	SBI-06-I	18.7	0-39	cdo456/ST1807	3.01	17.44	−5.535	*
QHGT_S2_7.1	SBI-07	18.8	14.8-33.6	txp312/FC20	1.68	3.69	−2.504	*
QHGT_S2_9.1	SBI-09	22.5	20.7-25	txs307b/txs1015	4.99	11.44	−4.581	**

^a^Peak position in cM based on the S2 genetic linkage map; ^b^The amount of total trait variance explained by a QTL at this locus, as %; ^c^The allelic effects are calculated as the effect of substitution of AA (ICSV745) allele by BB (R890562) allele; ^d^*Suggestive (LOD ≥ 2); **Significant (LOD ≥ 3); ***Highly significant (LOD ≥ 5).

**Table 4:** **Summary of rust resistance (QRustR), maturity (QDTF) and height (QHGT) QTL identified in the S4 population, detailing the QTL position, 2-LOD confidence interval (CI), flanking markers, peak LOD value, total trait variance explained (R** ^**2**^ **), additive effect, and significance level**

**QTL ID**	**LG**	**Peak** ^**a**^	**CI (cM)**	**Flanking markers**	**LOD**	**R** ^**2b**^	**Additive** ^**c**^	**Sig** ^**d**^
QRustR_S4_1.1	SBI-01	88.69	82.1-90	1892398|F|0/1885605|F|0	1.98	2.98	0.221	*
QRustR_S4_1.2	SBI-01	221.1	212.9-231.9	2651978|F|0/1957225|F|0	7.35	10.40	0.379	***
QRustR_S4_2.1	SBI-02	25.03	25.03-34.02	1945354|F|0/1935207|F|0	2.36	3.04	0.209	*
QRustR_S4_2.2	SBI-02	110.3	107.3-114	1942866|F|0/1944964|F|0	1.96	2.53	−0.190	*
QRustR_S4_3.1	SBI-03	82.74	74.05-86.3	1949016|F|0/1945627|F|0	4.41	6.07	−0.294	**
QRustR_S4_4.1	SBI-04	8.2	5.8-15.3	2207675|F|0/2663674|F|0	4.47	6.21	−0.296	**
QRustR_S4_4.2	SBI-04	76.97	74.7-78.9	1921138|F|0/2655283|F|0	2.03	2.93	−0.207	*
QRustR_S4_9.1	SBI-09	71.01	69-73.6	2657729|F|0/1950055|F|0	2.08	2.69	−0.220	*
QRustR_S4_10.1	SBI-10	160.9	153.4-166.5	1925698|F|0/2644849|F|0	3.81	5.21	−0.277	**
QDTF_S4_1.1	SBI-01	39.5	39.5	1923269|F|0/2645848|F|0	2.08	4.86	−0.567	*
QDTF_S4_2.1	SBI-02	139.4	131.0-142.7	2756003|F|0/1952851|F|0	2.17	5.43	0.578	*
QDTF_S4_3.1	SBI-03	151.6	150.6-152.5	2653022|F|0/2650636|F|0	2.63	6.40	0.638	*
QDTF_S4_6.1	SBI-06	20.6	16.6-29.8	2657488|F|0/1896474|F|0	6.09	23.08	−1.366	***
QDTF_S4_10.1	SBI-10	101.3	85.1-104.7	1905915|F|0/2656295|F|0	5.11	13.77	0.926	***
QHGT_S4_1.1	SBI-01	227.1	216.4-228.9	2653465|F|0/1944489|F|0	2.38	4.46	−3.498	*
QHGT_S4_4.1	SBI-04	28.9	28.9-31.9	2653424|F|0/2645563|F|0	2.24	4.75	3.819	*
QHGT_S4_5.1	SBI-05	49	48.5-54.8	2652538|F|0/2675950|F|0	2.68	5.96	−4.142	*
QHGT_S4_6.1	SBI-06	38.3	33.1-49.32	2650292|F|0/1919341|F|0	6.36	13.73	−6.344	***
QHGT_S4_7.1	SBI-07	75.1	63.4-83.5	1923401|F|0/2647631|F|0	5.53	12.65	−6.444	***
QHGT_S4_9.1	SBI-09	134.9	123.9-142.7	2648081|F|0/2652606|F|0	7.27	16.38	−6.913	***

^a^Peak position in cM based on the S4 genetic linkage map; ^b^The amount of total trait variance explained by a QTL at this locus, as %; ^c^The allelic effects are calculated as the effect of substitution of AA (IS8525) allele by BB (R931945-2-2) allele; ^d^*Suggestive (LOD ≥ 2); **Significant (LOD ≥ 3); ***Highly significant (LOD ≥ 5).

**Table 5:** **Summary of rust resistance QTL identified in the S7 population detailing the QTL location on consensus map, additive effect and significance level across 2 sites (BIL03** ^**IRR**^ **and BIL03** ^**nonIRR**^ **and the combined analysis, BIL03** ^**overall**^ **)**

			**Allele effects** ^**b**^		
**QTL ID**	**LG**	**Peak cM** ^**a**^	**BIL03** ^**IRR**^	**BIL03** ^**nonIRR**^	**BIL03** ^**overall**^
QRustR_S7_1.1	1	19.94	-0.170**	NS	NS
QRustR_S7_1.2	1	61.5	0.245**	NS	NS
QRustR_S7_1.3	1	151.33	-0.133**	NS	NS
QRustR_S7_2.1	2	144.02	0.195**	NS	NS
QRustR_S7_3.1	3	10.58	0.191**	NS	NS
QRustR_S7_3.2	3	107.3014	-0.225**	-0.122**	-0.119**
QRustR_S7_3.3	3	137.16	-0.129**	NS	NS
QRustR_S7_4.1	4	71.44	0.169***	0.148***	0.150***
QRustR_S7_4.2	4	82.8	0.303**	0.200***	0.202***
QRustR_S7_4.3	4	95.7	NS	0.122**	0.125**
QRustR_S7_5.1	5	75.65	-0.223**	NS	NS
QRustR_S7_7.1	7	131.58	NS	0.133**	NS
QRustR_S7_8.1	8	70.7	-0.221***	-0.165***	-0.159***
QRustR_S7_9.1	9	52.72	NS	-0.127**	-0.169**
QRustR_S7_9.2	9	87.27	NS	-0.145**	-0.146**
QRustR_S7_9.3	9	108.8	NS	-0.118**	-0.111**
QRustR_S7_10.1	10	59.84	NS	-0.124**	-0.121**
QRustR_S7_10.2	10	75.9	NS	-0.129**	-0.137***
QRustR_S7_10.3	10	103.29	-0.152**	NS	NS

^a^Peak position with maximum –log_10_P; ^b^The allelic effects are calculated as the effect of substitution of AA (R931945-2-2) allele by BB (*S. bicolor* subsp. *verticilliflorum*) allele. NS: not significant; **Significant (−log10P ≥ 3); ***Highly significant (−log10P ≥ 4).

### Rust resistance

In population S2, CIM identified two highly significant rust resistance QTL, one on SBI-05 and one on SBI-08-II (Table 3; Additional file 1: Figure S3). In addition, three suggestive QTL were detected on SBI-02, SBI-06-I and SBI-08-I. Individual QTL explained between 5.6 to 18.5% of phenotypic variation in response to rust, with a cumulative total of 53.7%. The majority of QTL (the three QTL on SBI-02, SBI-06-I and SBI-08-II) had positive allelic effects indicating that the ICSV745 QTL alleles predominately contributed to an increase in rust resistance.

In population S4, a total of 9 QTL for rust resistance were identified located on 6 chromosomes. One highly significant and three significant QTL were identified by CIM analysis on SBI-01, SBI-03, SBI-04 and SBI-10 (Table 4; Additional file 1: Figure S4). Five suggestive QTL were identified on SBI-01, SBI-02, SBI-04 and SBI-09. Individual QTL explained between 2.5 to 10.4% of phenotypic variation, with a cumulative total of 42.1%. Six of the 9 rust resistance QTL had negative effects, indicating that parent R931945-2-2 QTL alleles predominately contributed to an increase in rust resistance.

In population S7, fifteen genomic regions were detected with significant marker trait associations (*p* ≤ 0.001) on eight chromosomes (Table 5; Additional file 1: Figure S5). A further four genomic regions were detected with highly significant marker trait associations (*p* ≤ 0.0001). The majority (12/19) of the identified QTL had negative allele effects indicating that the *S. bicolor* subsp. *verticilliflorum* QTL alleles predominantly contributed to an increase in rust resistance.

**Figure 1:**
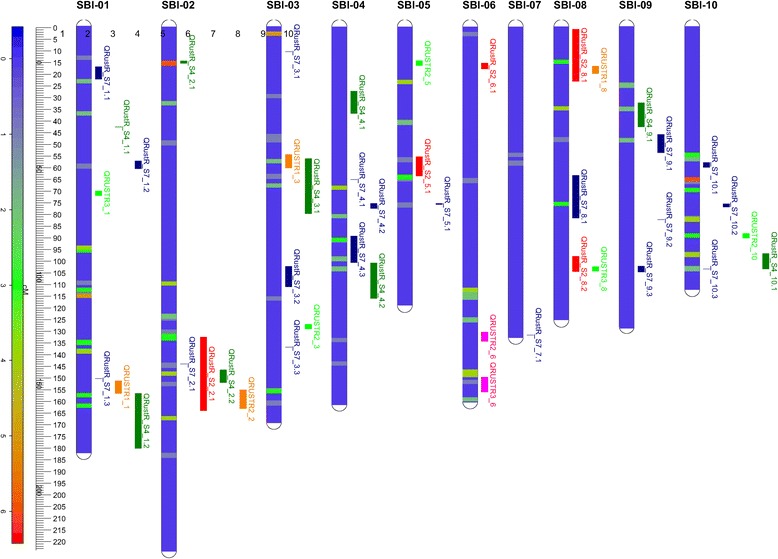
**Rust resistance QTL from the current study projected onto the sorghum consensus genetic linkage map in comparison with rust QTL identified in previous studies.** The bars to the right of each chromosome indicate rust resistance QTL identified, colour-coded as follows: red: S2 (current study); dark green: S4 (current study); dark blue: S7 (current study); green: [3]; orange; [5]; pink: [7]. The locations of the QTL identified in the AYT association mapping set are represented by a separate heat map, for each LG, detailing QTL density (number of QTL/0.5 cM).

In the AYT association mapping set, 52 genomic regions were identified with suggestive marker trait associations in at least one of the 6 tester/location combinations (*p* ≤ 0.0001), with over half (28) identified as significant in two or more of the tester/location combinations. To combine the results of the association mapping analyses across the six male tester and location combinations, the number of tester/location combinations with a significant marker trait association was calculated for each marker (Additional file 2: Table S4) and plotted against the sorghum consensus map based on a sliding window of 2 cM with a step size of 0.5 cM (Figure 1). Of the 52 QTL identified, 13 were identified in a single tester/location combination only and hence can be considered as suggestive QTL regions. Just over 10% (6/52) of the QTL were influenced by the genetic background, being identified only with specific male testers across both locations (e.g. QRustR_AYT_9.1 and QRustR_AYT_10.5 were identified only in combination with male tester R995248 across both sites). A further 21 QTL (40%) were location-specific, being identified only in one location, however these included the 13 suggestive QTL only identified in a single tester/location combination*.* Three QTL were identified in all three male tester combinations at a single site only (QRustR_AYT_1.8, QRustR_AYT_4.4, QRustR_AYT_6.4 identified in the Dalby site only). A further three QTL were identified in both sites and across all male tester combinations (QRustR_AYT_2.1, QRustR_AYT_6.5, QRustR_AYT_10.2). The total number of QTL identified with the male tester R986087-2-4-1, across both locations, was almost 50% higher than with either of the other two male testers, R993396 and R995248; 64 QTL vs 41 QTL vs 45 QTL respectively. The male line, R986087-2-4-1 was produced from a cross between R931945-2-2 and SC170-6-17; R931945-2-2 being a parental line of S4 and S7 populations. Of the 28 rust resistance QTL identified in both S4 and S7, 13 were derived from R931945-2-2. The graphical genotype of R986087-2-4-1 based on previously generated GBS data (data not shown) indicated that R986087-2-4-1 was identical by descent (IBD) to R931945-2-2 in 7 of these 13 QTL regions. In all 7 of these regions, a QTL was also identified in the AYT populations with the R986087-2-4-1 tester.

### Maturity

In the S2 population, five suggestive QTL for maturity were identified located on four chromosomes (SBI-03, SBI-04, SBI-05 and SBI-10), individually explaining between 6.2 to 10.4% of the phenotypic variation, with a cumulative total of 40.1% (Table 3; Additional file 1: Figure S3). The maturity QTL on SBI-05 co-located with the highly significant rust resistance QTL (QRustR_S2_5.1) in the same population (Χ^2^*p-*value <0.0001), with the R890562 alleles contributing towards lateness in maturity and rust resistance. In the S4 population, two highly significant QTL on SBI-06 and SBI-10 were identified, with a cumulative total of 36.9% of the phenotypic variation explained (Table 4; Additional file 1: Figure S4). Three additional suggestive QTL were identified on SBI-01, SBI-02 and SBI-03, individually explaining between 4.9 to 6.4% of the phenotypic variation. None of the maturity QTL co-located with rust QTL in the same population. Both positive and negative additive effects were observed, indicating that the R931945-2-2 parent had alleles contributing to both an increase and a decrease in maturity. In S7, 16 highly significant QTL were located on eight chromosomes (Additional file 2: Table S5). Fourteen of the 16 QTL had positive allele effects indicating that the *S. bicolor* subsp. *verticilliflorum* QTL alleles predominantly contributed to an increase in late maturity. A further two significant QTL regions were identified, both of which had positive allele effects. Overall, approximately a quarter (5/18) of the maturity QTL, four highly significant and one significant, in S7 co-located with rust QTL in the same population (Χ^2^*p-*value <0.0001). The direction of allelic effects was consistent across co-locating QTL, with the rust resistance QTL alleles from *S. bicolor* subsp. *verticilliflorum* always occurring with late maturing alleles from *S. bicolor* subsp. *verticilliflorum.* In the AYT association mapping set, 10 QTL for maturity were identified, eight of which co-located with rust QTL (Additional file 2: Table S6).

### Height

In the S2 population, one significant QTL on SBI-09 and three suggestive QTL on SBI-03, SBI-06 and SBI-07 were identified, with a cumulative total of 39.4% of phenotypic variation explained (Table 3; Additional file 1: Figure S3). Three of the four QTL had negative allele effects, indicating that the QTL alleles from R890562 predominantly contributed to a decrease in height. The height QTL on SBI-06 co-located with a suggestive rust QTL on SBI-06 (QRustR_S2_6.1) in the same population (Χ^2^*p-*value <0.0001), and the direction of QTL allele effects were consistent (i.e. the shorter plants carried the susceptible rust QTL allele). In the S4 population, three highly significant QTL for height were identified, with a cumulative total of 42.8% of the phenotypic variation explained, on chromosomes SBI-06, SBI-07 and SBI-09 (Table 4; Additional file 1: Figure S4). Three further suggestive QTL were identified on SBI-01, SBI-04 and SBI-05. The suggestive QTL for height on SBI-01 (QHGT_S4_1.1) co-located with a rust QTL (QRustR_S4_1.2) in the same population (Χ^2^*p-*value 0), while the highly significant height QTL on SBI-06 co-located with a maturity QTL. In both cases the direction of QTL allele effects was consistent with the observations from the S2 population (i.e. the susceptible rust QTL allele was associated with shorter and earlier alleles). In S7, only one highly significant QTL for height was identified on SBI-09, while a further 5 significant QTL were identified on SBI-03, SBI-04, SBI-06 and SBI-10 (Additional file 2: Table S7). Five of the height QTL identified had positive allele effects indicating that the *S. bicolor* subsp. *verticilliflorum* QTL alleles predominantly contributed to an increase in height. The significant QTL for height on SBI-10 (QHGT_S7_10.1) co-located with QTL for both rust and maturity, while the significant QTL for height on SBI-03 co-located within 10 cM a rust QTL (QRustR_S7_3.2) (Χ^2^*p-*value <0.0001). As previously, the direction of allele effects of the co-locating QTL was consistent with observations from the S2 and S4 populations (i.e. the taller and later maturing plants carried the rust resistant QTL allele). In the AYT association mapping set, 1 QTL for height was identified on SBI-09, which co-located with rust and maturity QTL also identified in the AYT population (Additional file 2: Table S6).

### Stay-green

Due to its association with carbon stress, the stay-green trait was also analysed in the AYT association mapping set. It was visually scored as a leaf senescence rating from 1–9 [23], across the same three male tester genotypes and two sites, with one trial site in common with the previous analyses, and analysed using the same methodology as described for the other traits, identifying nine significant QTL (Additional file 2: Table S8).

### QTL projection on consensus map

All the QTL identified in each of the three bi-parental populations and the AYT association mapping set were projected on the consensus map for all three traits (Figure 1; Additional file 1: Figures S6-7; Additional file 3: Table S9) and compared to rust, maturity and height QTL identified previously. Across the four populations in this study, 64 significant or highly significant QTL and 21 suggestive QTL for rust resistance were identified and projected onto the consensus map, in addition to the 11 previously reported QTL for rust resistance [3,5,7], representing a total of 56 unique regions (Additional file 4: Table S10). Rust QTL were identified on all chromosomes, however the genome-wide distribution was uneven, with only 2 unique QTL identified on SBI-07 in contrast to 15 rust QTL located on SBI-01. The syntenic locations of 52 rust QTL identified in maize [24-27] were also projected onto the consensus map and compared to the locations of the rust QTL from sorghum (Additional file 1: Figure S8). Ten of the previously reported sorghum rust QTL co-located with rust QTL identified in the current study; one genomic region described by [28] was found to be unique among the sorghum rust studies investigated, however this QTL co-located with maize rust QTL [25,27]. In total, of the 52 rust resistance QTL identified in maize, over 80% (42) co-located with rust QTL identified in sorghum (Additional file 1: Figure S8) (Χ^2^*p-*value <0.0001).

The 56 unique rust QTL regions identified were further categorised according to their co-location with height and maturity QTL, to distinguish between 1) QTL unique to rust within a population and 2) multi-trait effect QTL, with rust QTL co-locating with QTL for other traits within a population (Additional file 4: Table S10). Of the 56 unique genomic regions, 30 were categorised as being unique to rust, whereas 26 were not unique to rust, and co-located with either height, maturity or stay-green QTL identified in the same population. The correspondence of the two different types of rust resistance QTL locations and the NBS-LRR gene family members, previously reported to be associated with disease resistance [29], were investigated. The NBS-encoding genes were significantly enriched in the regions of the genome containing unique rust disease resistance QTL (Χ^2^*p-*value <0.0001), in contrast to the rust QTL that co-located with other traits (Χ^2^*p-*value 0.6118). In particular, the major NBS-encoding rust resistance gene *Rph1-2*, homologous with the maize *Rp1-D* gene, previously described in sorghum by [30] and also believed to be the *Pu* gene [13], co-located with QTL from the S2 population (QRustR_S2_8.1) and the AYT association mapping set (QRustR_AYT_8.1) (Additional file 1: Figure S9). Additionally, the wheat rust resistance gene, *Lr34*, previously described by [31], co-located with QTL from the S4 population (QRustR_S4_1.1) and the AYT association mapping set (QRustR_AYT_1.3) (Additional file 1: Figure S10), in addition to rust QTL identified in maize [25,26]. Both parental genotypes of the S4 and S7 populations were included in the recently described sorghum resequencing data set [32], enabling sequence analysis of underlying candidate genes. *In silico* analysis for missense mutations revealed a polymorphism leading to an amino-acid change that departed from the peptide arising from the predicted gene models [33] in the candidate gene for *Lr34* (Sb01g016775) which co-located with a rust resistance QTL in the S4 population (Additional file 1: Figure S11).

The thirty-eight QTL for maturity identified in S2, S4, S7 and the AYT association mapping set were projected onto the consensus map and compared to 157 maturity QTL reported previously. Of the 38 maturity QTL identified in the current study, 30 co-located with maturity QTL described previously (Additional file 1: Figure S6). Of the remaining 8 QTL, 6 co-located across the populations included in the current study, with only 2 novel maturity QTL identified (one in S7, QDTF_S7_8.3, and one in the AYT association mapping set, QDTF_AYT_9.2). Two major-effect maturity loci previously projected onto the sorghum consensus map [34] were identified in the current study; ma_1_ was identified in S4 (QDTF_S4_6.1), and ma_4_ was identified in populations S7 and S2 (QDTF_S7_10.1 and QDTF_S2_10.1). Similarly, the seventeen QTL for height identified in S2, S4, S7 and the AYT association mapping set were projected onto the consensus map and compared to 168 height QTL reported previously. Sixteen of the 17 height QTL identified co-located with height QTL previously described (Additional file 1: Figure S7), including the 3 major effect height loci previously projected onto the consensus map; dw_2_ on SBI-06 (QHGT_S4_6.1), dw_3_ on SBI-07 (QHGT_S4_7.1) and dw_1_ on SBI-09 (QHGT_S2_9.1 and QHGT_S4_9.1).

## Discussion

The rusts are a major pathogen of cereal crops with the potential to cause large reductions in yield and have as a result been the focus of ongoing plant breeding effort particularly in the winter cereals. The challenge for breeders in these species has been to produce stable resistance due to the rapidly evolving nature of the pathogen. Sorghum is a major summer cereal crop that is also a host for a rust pathogen *Puccinia purpurea*. Leaf rust occurs in almost all sorghum growing areas of the world [5] and can cause significant yield losses in the crop. In Australia, rust infection occurs in most sorghum crops with the severity of epidemics being greater in humid environments and in late-sown crops [8]. Despite the prevalence of the disease in sorghum, it is not subject to the large damaging rust epidemics which regularly occur in winter cereals in Australia.

Understanding the genetic basis of rust in sorghum will be important for improving the crop itself as well as providing a comparison and potential insight into the architecture of the trait in other species. This study used natural field infection data from multiple populations grown in multiple environments using a combination of conventional linkage mapping and association mapping to investigate the genetic control of rust resistance and the potential for multi-trait effect QTL.

### Rust resistance is a polygenic trait controlled by multiple QTL of small effect

The complex, polygenic nature of this trait was confirmed with 64 significant or highly significant QTL identified representing over 43 unique genomic regions, in addition to 21 suggestive QTL representing a further 12 unique genomic regions. Comparisons with previous rust resistance QTL studies revealed that 10 of the 11 previously reported rust resistance QTL in sorghum were also identified in the current study. The genetic architecture of the trait was largely found to involve multiple QTL of small effects, rather than major genes of large effect, with the largest allele effect reported in the S2 population (QRustR_S2_8.2 with an effect size of 0.56 units on a 1–9 scale), representing 18.4% of the total phenotypic variation, and with an average QTL allele effect across all QTL identified of just 0.23. This contrasts to the major effect leaf rust QTL commonly reported in other cereals, e.g. the major effect adult plant resistance gene *Rph20* in barley, where the additive allele effect sizes accounted for 64-85% of the phenotypic variation in adult plants across four field environments [35]; the major effect, “slow-rusting” resistance gene *Lr34* conferring durable adult plant resistance in wheat, explaining up to 55% of the phenotypic variation [36]. This difference in the genetic control of leaf rust resistance across cereals could be partly due to the fact that sorghum is primarily a perennial species in comparison to the strongly annual life-history of the other major cereals, where the need for more stable resistance due to increased plant longevity could favour selection for multiple resistance genes of small effect.

This study encompassed populations with a degree of shared ancestry, which enabled the evaluation of resistance alleles that were identical by descent (IBD) in different populations and experiments. The elite line R931945-2-2 was not only a common parent in the S4 and S7 populations but also a parent of the common male tester R986087-2-4-1 used to produce 150 of the F_1_ hybrids used in the association mapping set. QTL alleles from R931945-2-2 contributed to increased rust resistance in just under half (13/28) of the QTL identified from the two bi-parental populations; of these QTL, seven corresponded to genomic regions that were IBD between R986087-2-4-1 and R931945-2-2. In all 7 of these regions, a QTL was identified in this location in the AYT association mapping set, in hybrids with R986087-2-4-1 as the common tester. This result suggests that the inheritance of these seven resistance QTL is not completely dominant, but instead either additive or recessive. This conclusion is also supported by the detection of the vast majority (92%) of the significant QTL in the AYT association mapping set identified across multiple male tester hybrid combinations. In commercial agricultural settings sorghum cultivars are almost exclusively F_1_ hybrids and as result knowledge of the degree of dominance in traits is of critical importance for their deployment as cultivars.

The IBD analysis also enabled us to determine that two of the R931945-2-2 derived rust resistance QTL were identical to rust resistance QTL contributed by QL41 in the mapping population used by [5].The stability of these QTL suggests that there has been limited variation in the rust pathotypes over the last 15 years. In addition the fact that the majority of the rust QTL (80%) was detected at both AYT trial locations suggests that there is relatively low spatial and temporal variation in rust populations.

### Rust resistance is strongly influenced by the physiological state of the plant

In sorghum, rust infection generally develops later in the crop’s cycle as the plant begins filling grain [10]. This is consistent with the observation of [17] who demonstrated that the growth stage, or physiological age, of groundnut (*Arachis hypogaea* L.) can impact on the degree of susceptibility to rust infection. Typically plants in vegetative stages are less susceptible to many diseases than those infected at reproductive phases, when the plant is remobilizing carbohydrates during grain-filling [37]. This phenomenon has important implications for assessing rust resistance in plants that vary in maturity or other characters such as height or stay-green which influence the developmental status of the plant and hence the nature and degree of carbohydrate remobilization. This is particularly important in field grown plants subject to natural infection. Although the association between height and rust response could be related to a variation in microclimate, the association is consistent with the change in source/sink dynamics where earlier maturing plants become vulnerable to infection before later plants and taller plants have greater reserves for remobilization. Carbon stress also plays an important role in drought responsiveness. Plants with the stay-green characteristics have increased functional carbohydrate resources for remobilisation during grain filling [38].

We observed low but consistent, negative phenotypic correlations between the severity of rust infection and both maturity and height. In all populations and trials early maturity and short stature was associated with rust susceptibility. This result is consistent with the hypothesis that variation in plant carbohydrate status influences susceptibility to rust. At the QTL level we found a highly significant coincidence of rust QTL with QTL for height and maturity in the four populations used. A statistically significant proportion (~37%; Χ^2^*p-*value <0.0001) of the rust QTL co-located with one of these traits in the same population. A recent synthesis of disease resistance QTL studies in maize found comparable significant associations between disease resistance QTL and maturity QTL in the maize genome [39].

Co-locating QTL regions could contain multiple tightly linked genes for resistance and other traits or single genes with a pleiotropic effect across multiple traits. However the evidence for pleiotropic QTL action in the current study is very strong given the consistency in the direction of QTL allele effects, i.e. taller and later maturing plants carried the rust resistant QTL allele in the majority of cases (>80%). This is consistent with a recent study analysing the genetic control of rust in sorghum [3] who found rust severity in field grown sorghum was negatively correlated with plant height and maturity. Interestingly, when the same genotypes were artificially infected at a single time point in the glasshouse these correlations were not apparent [3]. This provides further evidence that the physiological state influences either the timing of infection or the rate at which infection develops.

The co-location of 100% of the leaf senescence, stay-green characteristic, QTL identified in the AYT association mapping set with rust QTL, in addition to the consistency of the direct of the effects (i.e. lines with alleles for increased senescence were more susceptible to rust), provides further evidence to support the role of carbon stress in disease resistance. Additionally, when all 96 rust QTL were compared to the location of 83 QTL for stay-green reported in seven previous studies [23,40-45], there was significant co-location across traits (Χ^2^*p-*value <0.0001) with over half of the 96 rust QTL co-locating with stay-green QTL. The highly significant QTL coincidence between rust and stay-green suggests many QTL impacting on both traits through a pleiotropic effect on carbon mobilisation.

### Rust QTL that are not associated with maturity and height are enriched for defence-related gene families

Gene families associated with defence and disease resistance have previously been reported; the NBS-LRR gene family being the most prevalent and ancient and one of the largest gene families known in plants to be involved in the detection and response to diverse pathogens, including bacteria, viruses, fungi, nematodes, insects and oomycetes [46]. The current study found that rust resistance QTL that were unique, and did not co-locate with either height, maturity or stay-green QTL (to be termed classical rust resistance QTL), were significantly enriched for defence-related gene families (Χ^2^*p-*value <0.0001), in contrast to the rust resistance QTL with potential pleiotropic effect that co-located with either height, maturity or stay-green QTL (Χ^2^*p-*value 0.6118) (to be termed multi-trait effect QTL).

### Some sorghum rust QTL fall within hotspots for multiple disease resistance

High correlations between resistances to rust and other biotic stresses have been reported previously in sorghum such as the positive correlation between resistance to rust, target leaf spot, zonate leaf spot and anthraconose observed by [7]. Additionally, hot-spots in the genome for multiple disease resistances are present in sorghum, particularly the short arm of SBI-10 and the long arm of SBI-06, containing 51 QTL for 12 traits from 10 additional studies, as well as the current study [3,7,47-54]. These hotspots could be the result of genes which provide resistance to multiple diseases, clusters of resistance genes or physiological pleiotrophy driven by regulation of carbon mobilisation.

Of the 85 significant and suggestive rust QTL reported in the current study, over three quarters (65/85) co-located with 178 sorghum pest and disease resistance QTL from 23 studies previously projected onto the consensus map [22] (Additional file 5: Table S11). Particular types of pest and disease resistance QTL were more likely to co-locate with the rust QTL associated with physiological pleiotrophic effects (e.g. ergot resistance, green bug and shoot fly resistance QTL) while the reverse was true of fungal pathogens of the leaf (anthracnose and zonate leaf spot) which predominantly co-locating with the classical rust resistance QTL, which did not co-locate with QTL associated with carbon stress.

The two stand-out hotspot regions identified for multiple disease resistances on SBI-10 and SBI-06 contained NBS-LRR gene clusters. Our result is consistent with three previous studies in sorghum that reported NBS-LRR genes conferring resistance to fungal pathogens; St-R gene cluster conferring resistance to *Setosphaeria turcica* causing northern leaf blight in maize [55], Cs1 and Cs2 genes conferring resistance to *Colletotrichum sublineolum* causing anthracnose [56] and *Rph*1-2 conferring resistance to leaf rust [3,57].

## Conclusions

This study has dissected the genetic architecture of rust resistance in sorghum, through conventional QTL mapping and association mapping, with multiple rust resistance QTL of small effect identified. Using multiple lines of evidence, including colocation of QTL, direction of QTL effects and disease resistance gene family enrichment, we were able to classify 54 rust QTL identified in the current study as classical rust resistance QTL, and 31 rust QTL as multi-trait effect QTL where susceptibility is likely to have resulted from genes that influence changes in the plant carbon mobilisation during the reproductive phase. The results of this study have significant implications for breeding programs attempting to increase disease resistance, particularly using molecular breeding approaches, because of the danger of inadvertently imposing selection pressure on associated traits, such as height and maturity, rather than disease resistance *per se*.

The genetic architecture of rust resistance in sorghum contrasts to rust resistance genes of large effect identified in other cereals, such as wheat, barley and oats, where rust epidemics frequently have more severe yield costs. The depletion of plant carbon in the determinate annual winter cereal species, in contrast to the increased tendency towards perenniality in sorghum, could impose more severe carbon stress in wheat, barley and oats. Understanding of how senescence or other drivers of carbon remobilisation increase susceptibility to leaf diseases may provide a useful avenue for future work on developing novel approaches to controlling these diseases in other cereals.

## Methods

### Mapping populations

Three sorghum bi-parental populations, developed by the Department of Agriculture, Forestry and Fisheries (DAFF) were investigated in this study and consisted of either F_2_-based recombinant inbred lines (RILs) or BC_1_F_1_ derived RILs. The first population consisted of 119 F_5_ RILs developed from the cross between ICSV745 and R890562-1-2, coded S2. The second population (S4) consisted of 246 F_5_ RILs derived from the IS8525/R931945-2-2 cross as described by [58]. ICSV745 and R931945-2-2 showed partial resistance to rust infection (Jordan, pers. comm.), whereas IS8525 and R890562-1-2 were susceptible to rust infection. The third population (S7) consisted of 214 backcross derived (BC_1_F_4_) RILs, derived from crossing *S. bicolor* subsp. *verticilliflorum* (a wild sorghum, accession number in AusPGRIS: AusTRCF 317961) and the recurrent parent R931945-2-2. In the current study, the S7 population was evaluated in hybrid combination with a single female tester (B923171). An additional set of 150 genotypes (Additional file 6: Table S12), consisting of elite female parent lines in the advanced yield testing (AYT) stage of the sorghum pre-breeding program, were evaluated in hybrid combination with three male testers (R986087-2-4-1, R993396, R995248).

### Field trials and phenotypic screens

The S2 and S4 populations were planted in fully replicated, single-row plots (5.5 m × 0.7 m) at the Hermitage Research Facility (HRF, latitude −28.167, long. 152.033, altitude 480 m asl), Warwick, Queensland, Australia during the 2009–2010 growing season (HRS10). The S7 population was grown under two environments: irrigation (BIL2003^IRR^) and dry (BIL2003^nonIRR^) at Biloela (latitude −24.400, long. 150.513, altitude 193 m asl) in 2003, both with fully replicated designs. The AYT female parents were grown at two different locations (Dalby, latitude −27.181, long. 151.266, altitude 341 m asl; Dalby2011) and Liverpool Plains (latitude −31.267, long. 150.047, altitude 308 m asl; Liverpool Plains2011) during the 2011–2012 growing season and in hybrid combination with three different male tester genotypes. The AYT trials were partially replicated, with 30% replication.

The trials reported in these studies made use of natural field infection rather than artificial inoculation with known strains. Rust infection was visually scored using a 1–9 scale with 1 indicating immune and 9 highly susceptible [5]. Plots were deemed to have flowered when 50% of the plants in the plot had started flowering [59]. The number of days to flowering (DTF) was surveyed as the days needed from sowing to flowering, and height (HGT) was determined at maturity from the soil surface at the base of the culm to the top of the panicle.

### Statistical analysis of the trait data

For all experiments a linear mixed model was fitted to the raw data with genotype as a fixed effect and replicate as a random effect. Each experiment S2, S4, S7 and AYT were analysed separately and spatial terms were added where necessary to accommodate for all possible errors due to trial design and field layout. ASReml-R [60] used the residual maximum likelihood (REML) algorithm to provide best linear unbiased estimates (BLUEs) as the predicted values for the breeding lines. The predicted BLUEs were used for association mapping.

### Linkage map construction

For the S2 population, existing genotypic data was used, as described previously [21], consisting of 488 markers (DArTs, RFLPs and SSRs). For the S4 and S7 populations, total genomic DNA of the progeny was extracted from two week old seedlings as described by [61]. The samples were genotyped with DArTseq™ technology, which represents a combination of DArT complexity reduction methods based on methyl filtration and next-generation sequencing platforms [62]. In total, 4091 polymorphic SNP markers were identified in the S4 population and 9545 polymorphic SNP markers in the S7 population. Genetic linkage maps were constructed for the S2 and S4 populations using MultiPoint, as described previously [21], resulting in framework maps consisting of 261 markers mapped to 12 linkage groups (1317 cM total length) for the S2 population and 964 markers mapped to 10 linkage groups (1476 cM total length) for the S4 population. Due to the backcross nature of the S7 population, in addition to selection applied for maturity during population development, the standard genetic linkage mapping approaches typically applied to bi-parental crosses were not appropriate. Instead the physical base pairs locations of the SNP markers were used to predict the location of each marker on the consensus genetic linkage map of sorghum [21] using a framework map of sequenced markers on the consensus map as detailed in [22].

### QTL analysis

QTL analyses of rust, height and maturity were conducted for the two RIL mapping populations using composite interval mapping (CIM) in QTL Cartographer for Windows v2.5 [63]. Background markers for inclusion in the CIM model were selected by forward stepwise regression for each trait. The five most significant background markers were then used for analysis (default). The ‘walking speed’ was set at 2 cM and the ‘window size’ at 10 cM for CIM. A conservative permutation threshold at the 0.01 significance threshold was obtained for each trait using 1,000 permutations. 1-LOD and 2-LOD support intervals were determined as described by [64]. The additive effects and percentage of variation explained (R^2^) for all significant QTL were determined at their peak LOD values. The significance level of each trait in the two RIL mapping populations was obtained by permutation analyses using Map Manager QTX software [65]. For the S7 population, standard QTL mapping approaches were not appropriate due to its back-cross derived nature. Hence, a mixed model marker-trait association analysis was performed using the association mapping function in GenStat [66]. The same analysis methodology was used to analyse the AYT association mapping set. Population structure was accounted for using the eigenanalysis relationship model. The Wald statistical test was used for each marker to test the null hypothesis that the marker’s effect was zero. Linkage Disequilibrium (LD) decay was determined in the two germplasm sets to determine significance thresholds to apply (Additional file 1: Figure S12). The following QTL nomenclature was adopted for this study: the prefix “Q”, followed by the abbreviated trait name, population code, chromosome and a final number suffix indicating the QTL number per chromosome.

### QTL projection onto a consensus map

To date, 11 rust resistance QTL in sorghum have been reported in three previous studies [3,5,7] (Table 6). The four rust QTL identified by [5] were included in the previous study by [22], which reported on the projected locations of 771 sorghum QTL from 44 studies onto the sorghum consensus genetic linkage map. The remaining 7 rust QTL previously reported were also projected onto the consensus genetic linkage map, following the strategy detailed by [22]. Additionally 168 QTL for height were also projected onto the consensus genetic linkage map, and included 101 QTL from 15 studies previously detailed in [22] and an additional 67 QTL from 7 more recent studies [28,67-72] (Additional file 3: Table S9). Similarly, 157 QTL for maturity were projected onto the consensus genetic linkage map, and included 62 QTL from 12 studies previously detailed in [20] and an additional 95 QTL from 8 more recent studies [28,59,67,68,70-73] (Additional file 3: Table S9). The QTL for all three traits identified in the three separate mapping populations in the current study were also projected onto the sorghum consensus map, based on the physical locations of the flanking markers, to facilitate comparisons across populations, studies and traits. Finally, the syntenic locations of rust resistance QTL from maize, as detailed in [27], were identified in sorghum using comparative genomics resources developed by [74].

**Table 6:** **Details of three previously published rust resistance QTL studies in sorghum including population pedigree and size, generation, number of QTL identified, and analysis method used (IM: interval mapping, NPM: non-parametric mapping, CIM: composite interval mapping, MLM: mixed linear model)**

**Publication**	**Pedigree**	**Pop size**	**Generation**	**No. QTL**	**Analysis method**
[3]	Mini-core	242	-	5	MLM
[5]	QL39/QL41	160	RILs*	4	IM, NPM
[7]	296B/IS18551	168	RILs*	2	IM, CIM

*RILs: recombinant inbred lines.

The significance of the degree of co-location of QTL (defined as two QTL either having overlapping CI or the mean of the QTL being less than 10 cM apart) 1) across traits within populations, 2) of rust QTL in sorghum and maize, 3) with previously identified NBS-LRR genes in sorghum [75] and 4) of rust and stay-green QTL in sorghum were determined using chi-square statistics, assuming random distribution of QTL genome-wide.

### Availability of supporting data

The data supporting the results of this article are included as Additional files 1, 2, 3, 4, 5, 6.

## Additional files

Additional file 1:
**Contains 12 supplementary figures, all referenced in the main text.**
Additional file 2:
**Contains 8 supplementary tables, all referenced in the main text.**
Additional file 3: Table S9. Details of all rust, height and maturity QTL projected onto consensus map. Additional file 4: Table S10. List of the 56 meta rust resistance QTL, with details of co-location with height, maturity and stay-green (leaf senescence) QTL, maize rust resistance QTL and NBS-encoding genes. Additional file 5: Table S11. Details of the disease resistance QTL identified in 23 previous studies. Additional file 6: Table S12. Details of the 150 elite female parent lines and the rust disease scores in hybrid combination with the three male testers (R986087-2-4-1, R993396, R995248).

## Abbreviations

QTL: Quantitative trait loci
RILs: Recombinant Inbred Lines
AYT: Advanced yield testing
IBD: Identity by descent
GBS: Genotyping-by-sequencing
NBS-LRR: Nucleotide-binding site plus leucine-rich repeat
