# Supplementary material for: Two distinct classes of QTL determine rust resistance in sorghum
Source: BMC Plant Biol. 2014 Dec 31;14:366. doi: 10.1186/s12870-014-0366-4 (PMC4335369; doi:10.1186/s12870-014-0366-4)
Supplement: Additional file 1: — Contains 12 supplementary figures, all referenced in the main text. [file 12870_2014_366_MOESM1_ESM.ppt]

## Slide 1
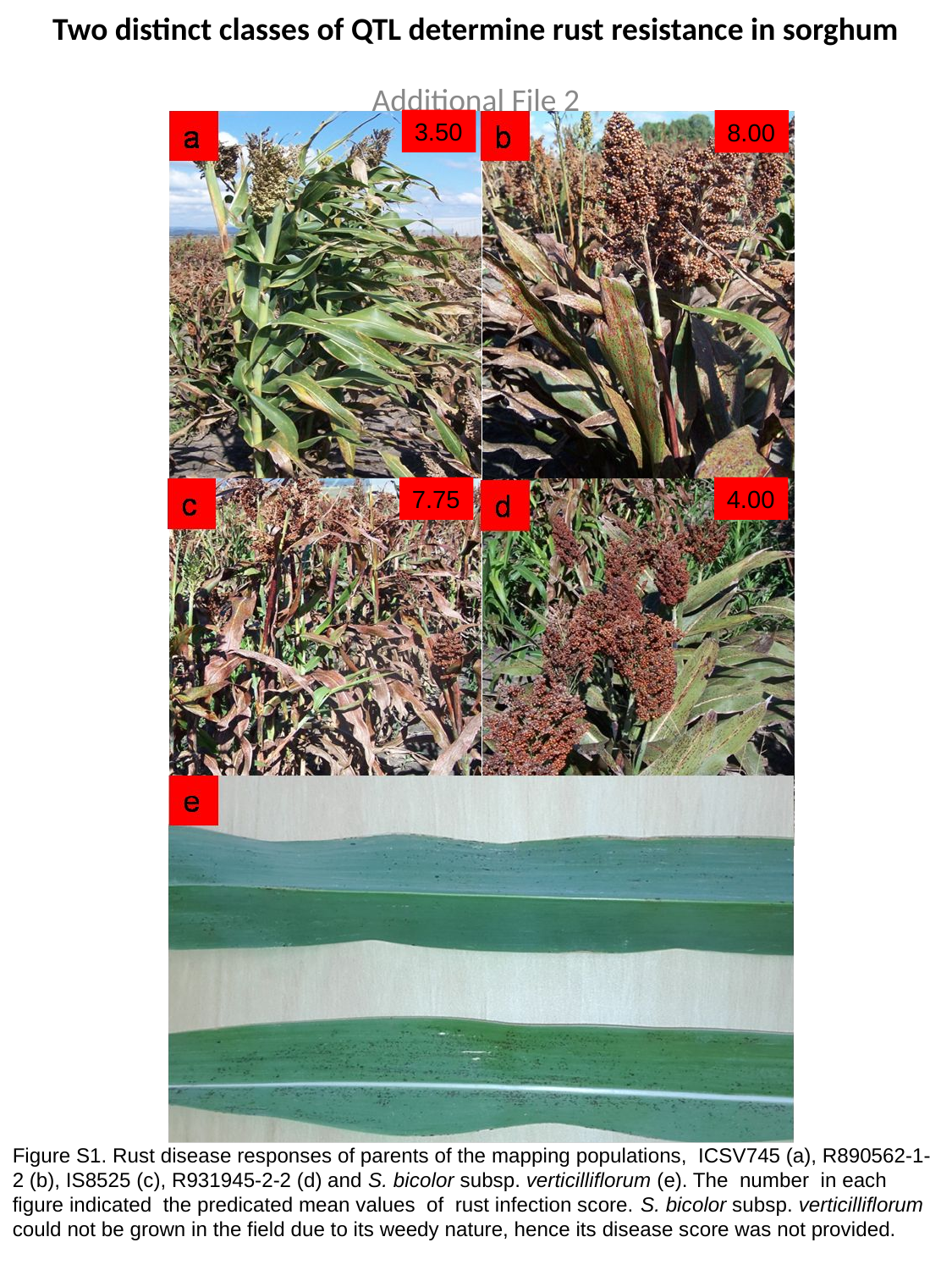

Two distinct classes of QTL determine rust resistance in sorghum
Additional File 2
3.50
8.00
7.75
4.00
# Figure S1. Rust disease responses of parents of the mapping populations, ICSV745 (a), R890562-1-2 (b), IS8525 (c), R931945-2-2 (d) and S. bicolor subsp. verticilliflorum (e). The number in each figure indicated the predicated mean values of rust infection score. S. bicolor subsp. verticilliflorum could not be grown in the field due to its weedy nature, hence its disease score was not provided.

## Slide 2
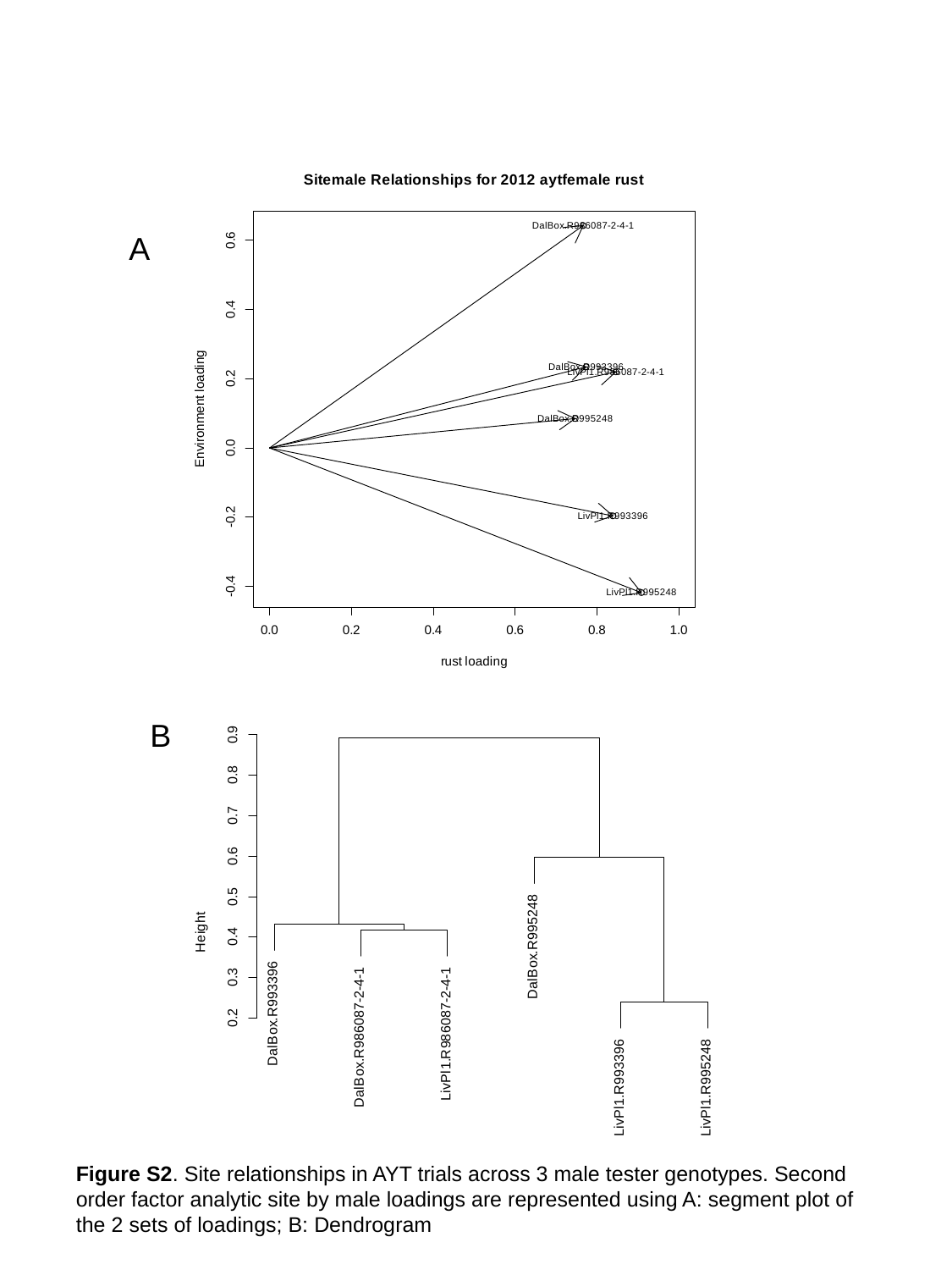

A
B
Figure S2. Site relationships in AYT trials across 3 male tester genotypes. Second order factor analytic site by male loadings are represented using A: segment plot of the 2 sets of loadings; B: Dendrogram

## Slide 3
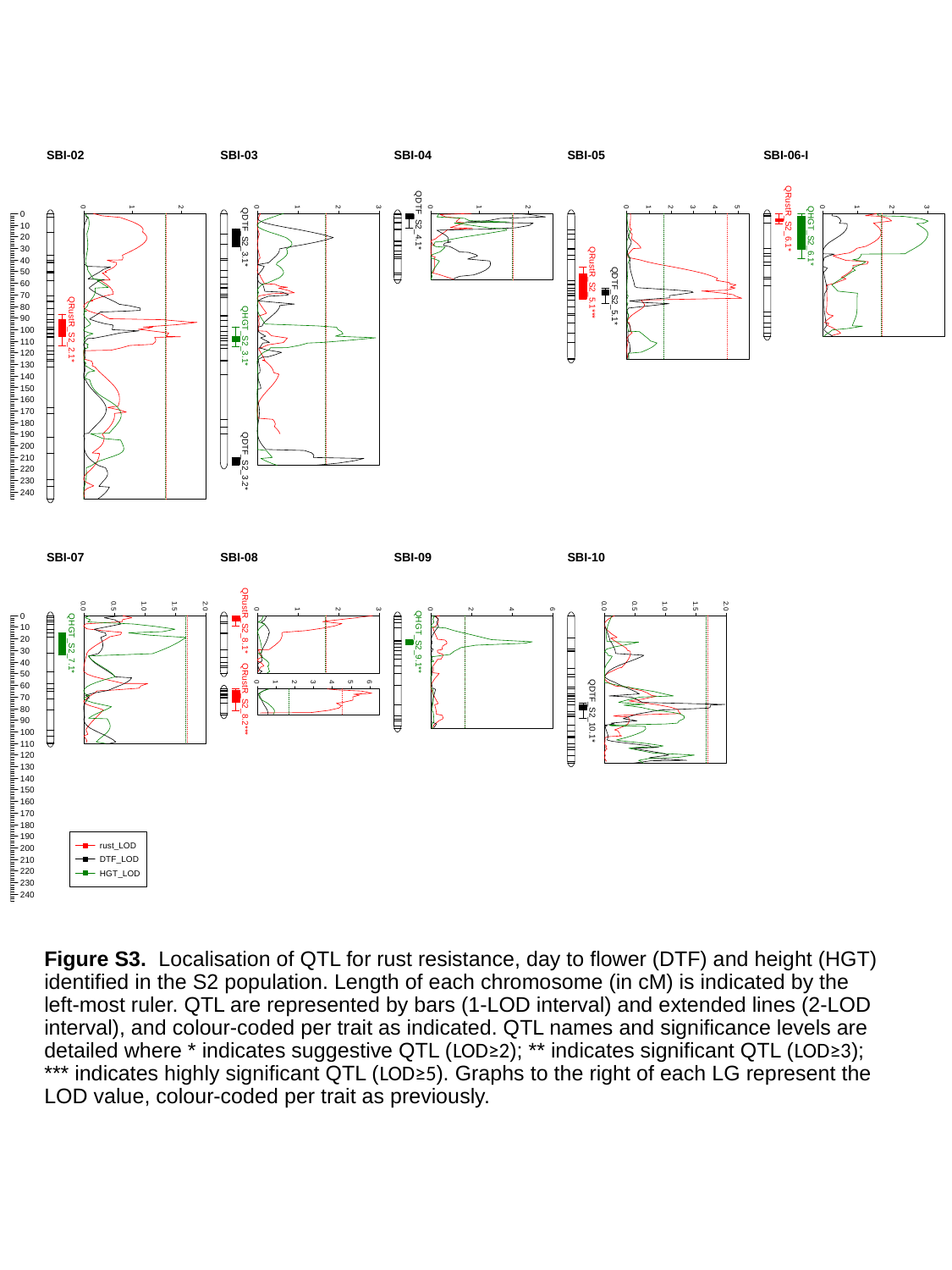

# Figure S3. Localisation of QTL for rust resistance, day to flower (DTF) and height (HGT) identified in the S2 population. Length of each chromosome (in cM) is indicated by the left-most ruler. QTL are represented by bars (1-LOD interval) and extended lines (2-LOD interval), and colour-coded per trait as indicated. QTL names and significance levels are detailed where * indicates suggestive QTL (LOD≥2); ** indicates significant QTL (LOD≥3); *** indicates highly significant QTL (LOD≥5). Graphs to the right of each LG represent the LOD value, colour-coded per trait as previously.

## Slide 4
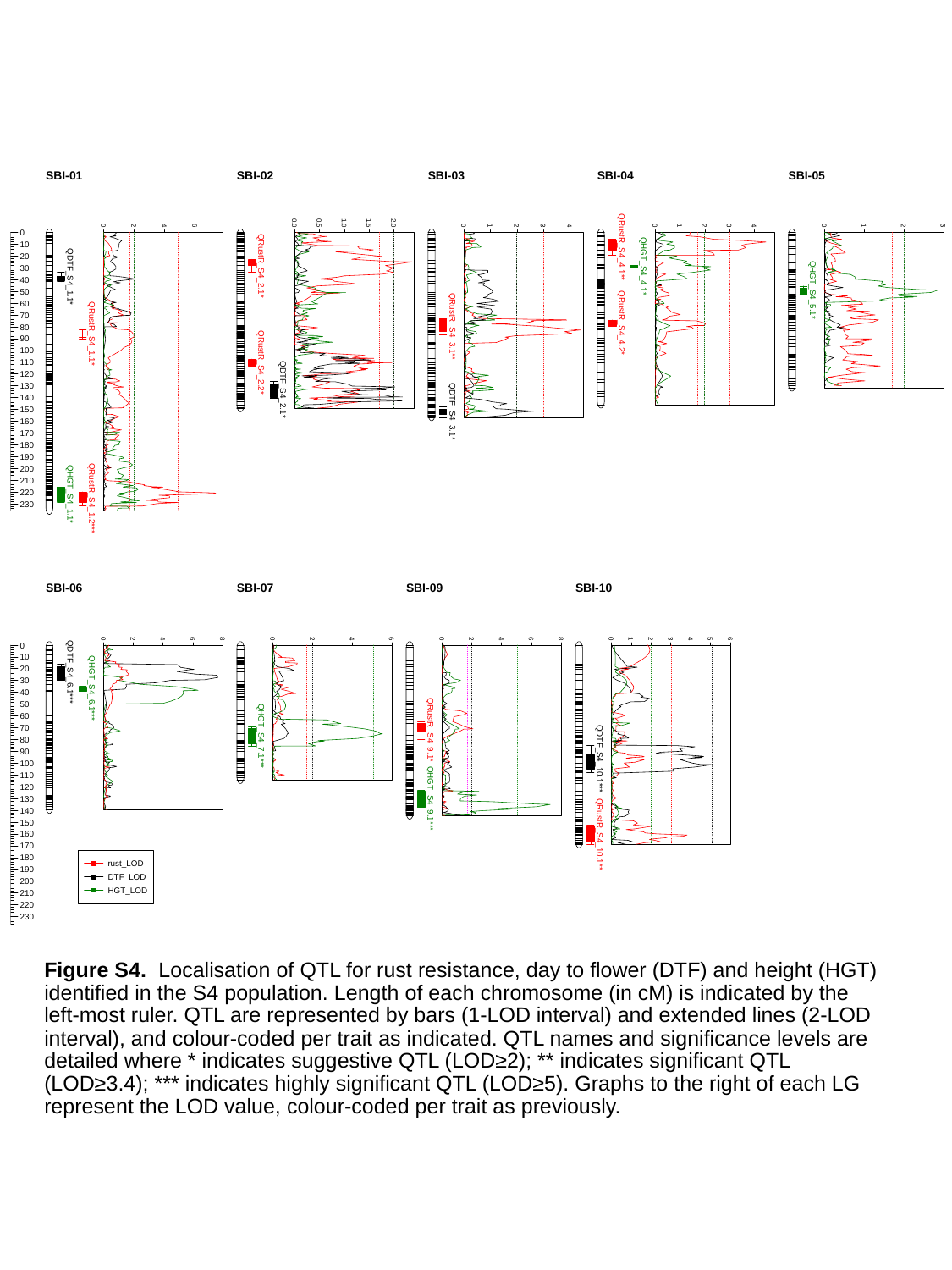

Figure S4. Localisation of QTL for rust resistance, day to flower (DTF) and height (HGT) identified in the S4 population. Length of each chromosome (in cM) is indicated by the left-most ruler. QTL are represented by bars (1-LOD interval) and extended lines (2-LOD interval), and colour-coded per trait as indicated. QTL names and significance levels are detailed where * indicates suggestive QTL (LOD≥2); ** indicates significant QTL (LOD≥3.4); *** indicates highly significant QTL (LOD≥5). Graphs to the right of each LG represent the LOD value, colour-coded per trait as previously.

## Slide 5
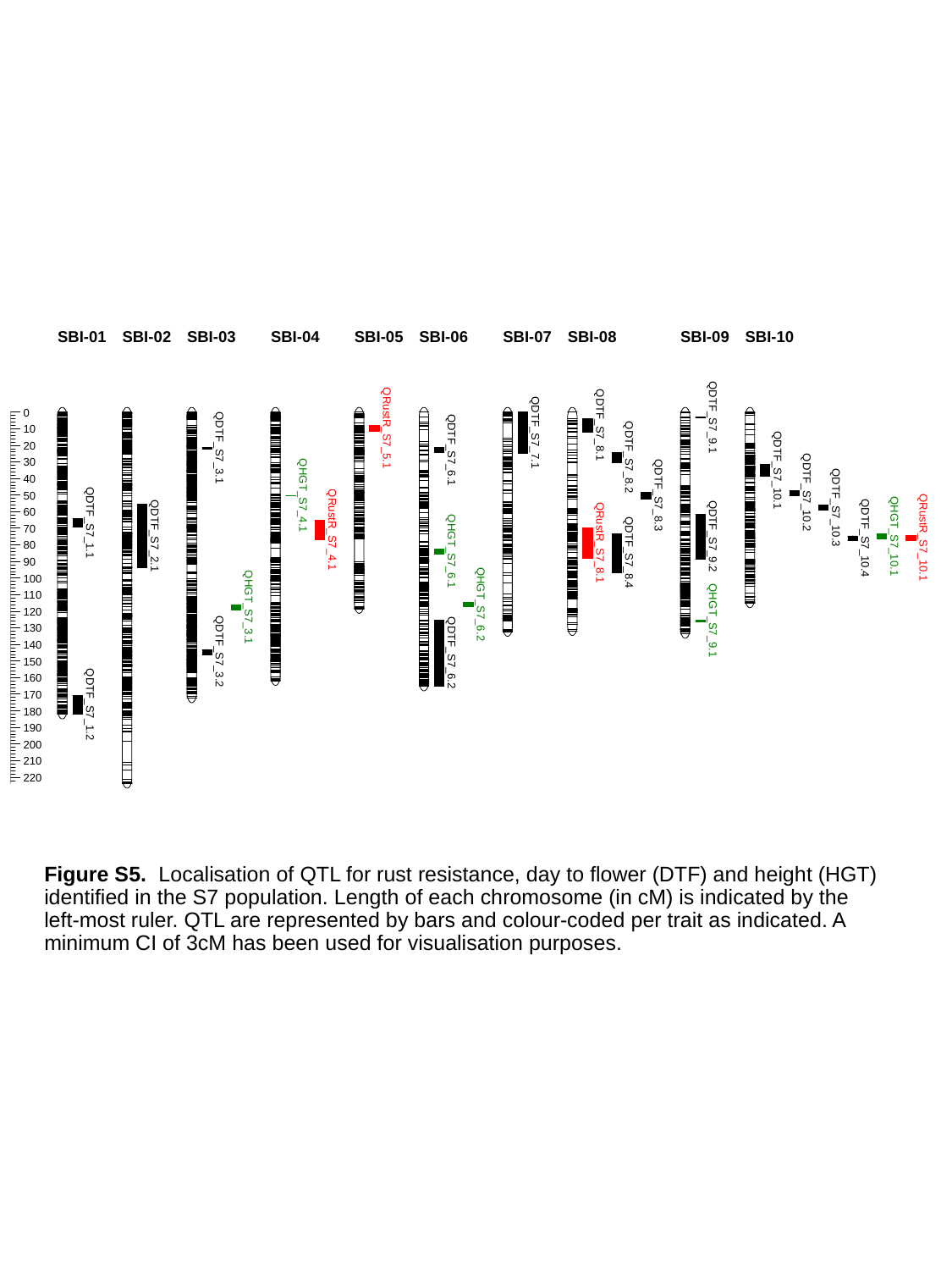

Figure S5. Localisation of QTL for rust resistance, day to flower (DTF) and height (HGT) identified in the S7 population. Length of each chromosome (in cM) is indicated by the left-most ruler. QTL are represented by bars and colour-coded per trait as indicated. A minimum CI of 3cM has been used for visualisation purposes.

## Slide 6
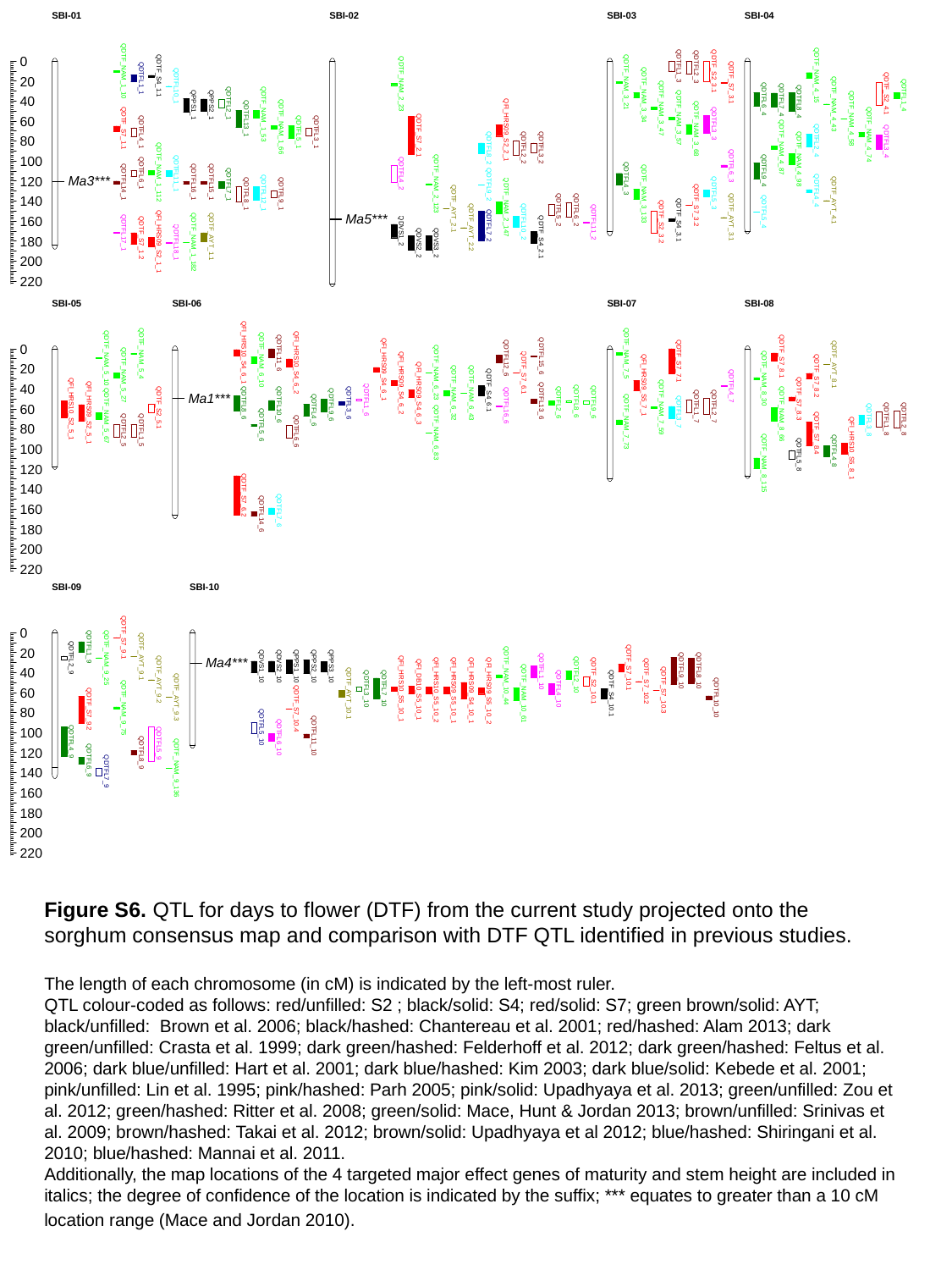

# Figure S6. QTL for days to flower (DTF) from the current study projected onto the sorghum consensus map and comparison with DTF QTL identified in previous studies. The length of each chromosome (in cM) is indicated by the left-most ruler. QTL colour-coded as follows: red/unfilled: S2 ; black/solid: S4; red/solid: S7; green brown/solid: AYT; black/unfilled: Brown et al. 2006; black/hashed: Chantereau et al. 2001; red/hashed: Alam 2013; dark green/unfilled: Crasta et al. 1999; dark green/hashed: Felderhoff et al. 2012; dark green/hashed: Feltus et al. 2006; dark blue/unfilled: Hart et al. 2001; dark blue/hashed: Kim 2003; dark blue/solid: Kebede et al. 2001; pink/unfilled: Lin et al. 1995; pink/hashed: Parh 2005; pink/solid: Upadhyaya et al. 2013; green/unfilled: Zou et al. 2012; green/hashed: Ritter et al. 2008; green/solid: Mace, Hunt & Jordan 2013; brown/unfilled: Srinivas et al. 2009; brown/hashed: Takai et al. 2012; brown/solid: Upadhyaya et al 2012; blue/hashed: Shiringani et al. 2010; blue/hashed: Mannai et al. 2011. Additionally, the map locations of the 4 targeted major effect genes of maturity and stem height are included in italics; the degree of confidence of the location is indicated by the suffix; *** equates to greater than a 10 cM location range (Mace and Jordan 2010).

## Slide 7
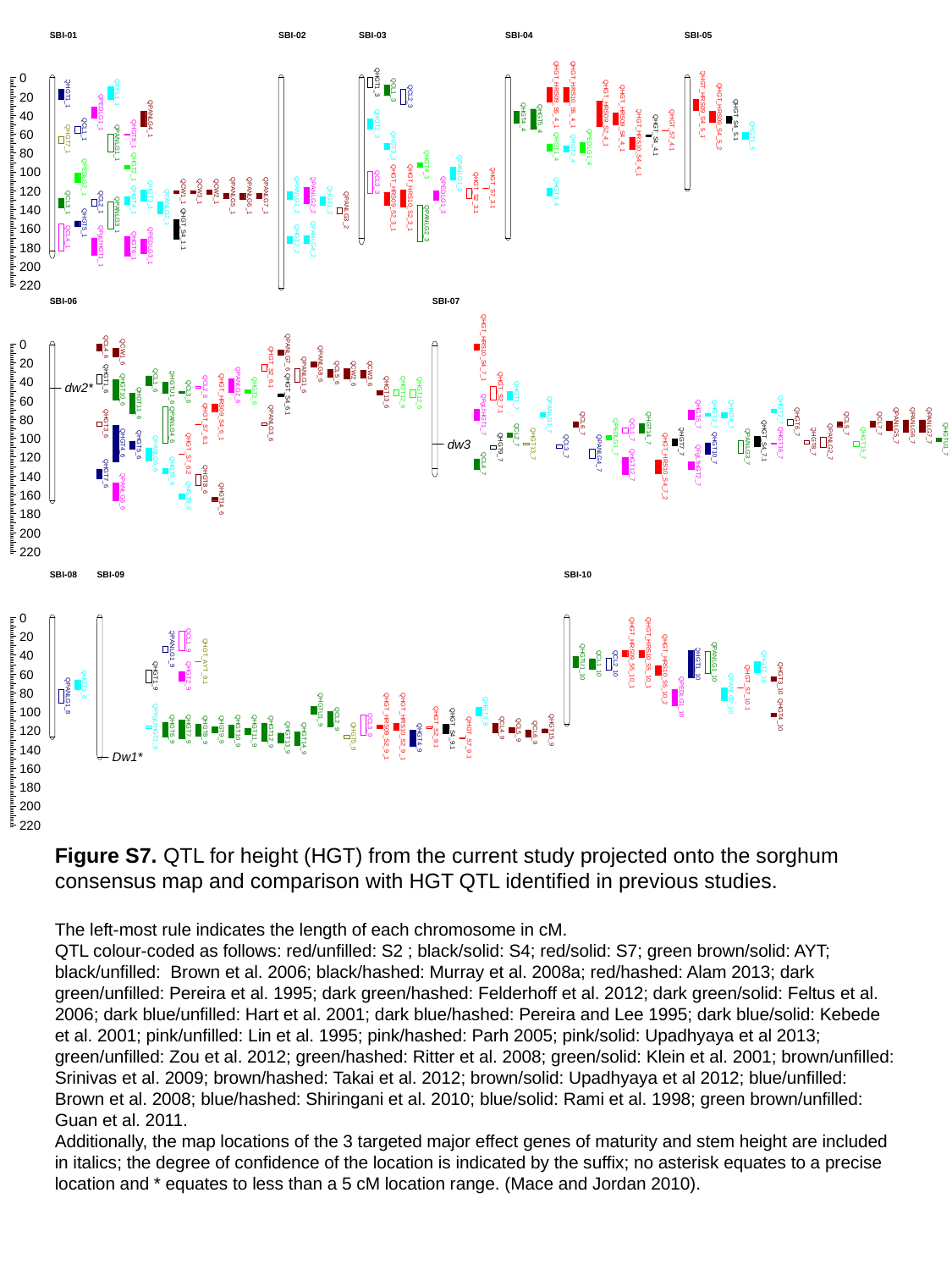

# Figure S7. QTL for height (HGT) from the current study projected onto the sorghum consensus map and comparison with HGT QTL identified in previous studies. The left-most rule indicates the length of each chromosome in cM. QTL colour-coded as follows: red/unfilled: S2 ; black/solid: S4; red/solid: S7; green brown/solid: AYT; black/unfilled: Brown et al. 2006; black/hashed: Murray et al. 2008a; red/hashed: Alam 2013; dark green/unfilled: Pereira et al. 1995; dark green/hashed: Felderhoff et al. 2012; dark green/solid: Feltus et al. 2006; dark blue/unfilled: Hart et al. 2001; dark blue/hashed: Pereira and Lee 1995; dark blue/solid: Kebede et al. 2001; pink/unfilled: Lin et al. 1995; pink/hashed: Parh 2005; pink/solid: Upadhyaya et al 2013; green/unfilled: Zou et al. 2012; green/hashed: Ritter et al. 2008; green/solid: Klein et al. 2001; brown/unfilled: Srinivas et al. 2009; brown/hashed: Takai et al. 2012; brown/solid: Upadhyaya et al 2012; blue/unfilled: Brown et al. 2008; blue/hashed: Shiringani et al. 2010; blue/solid: Rami et al. 1998; green brown/unfilled: Guan et al. 2011. Additionally, the map locations of the 3 targeted major effect genes of maturity and stem height are included in italics; the degree of confidence of the location is indicated by the suffix; no asterisk equates to a precise location and * equates to less than a 5 cM location range. (Mace and Jordan 2010).

## Slide 8
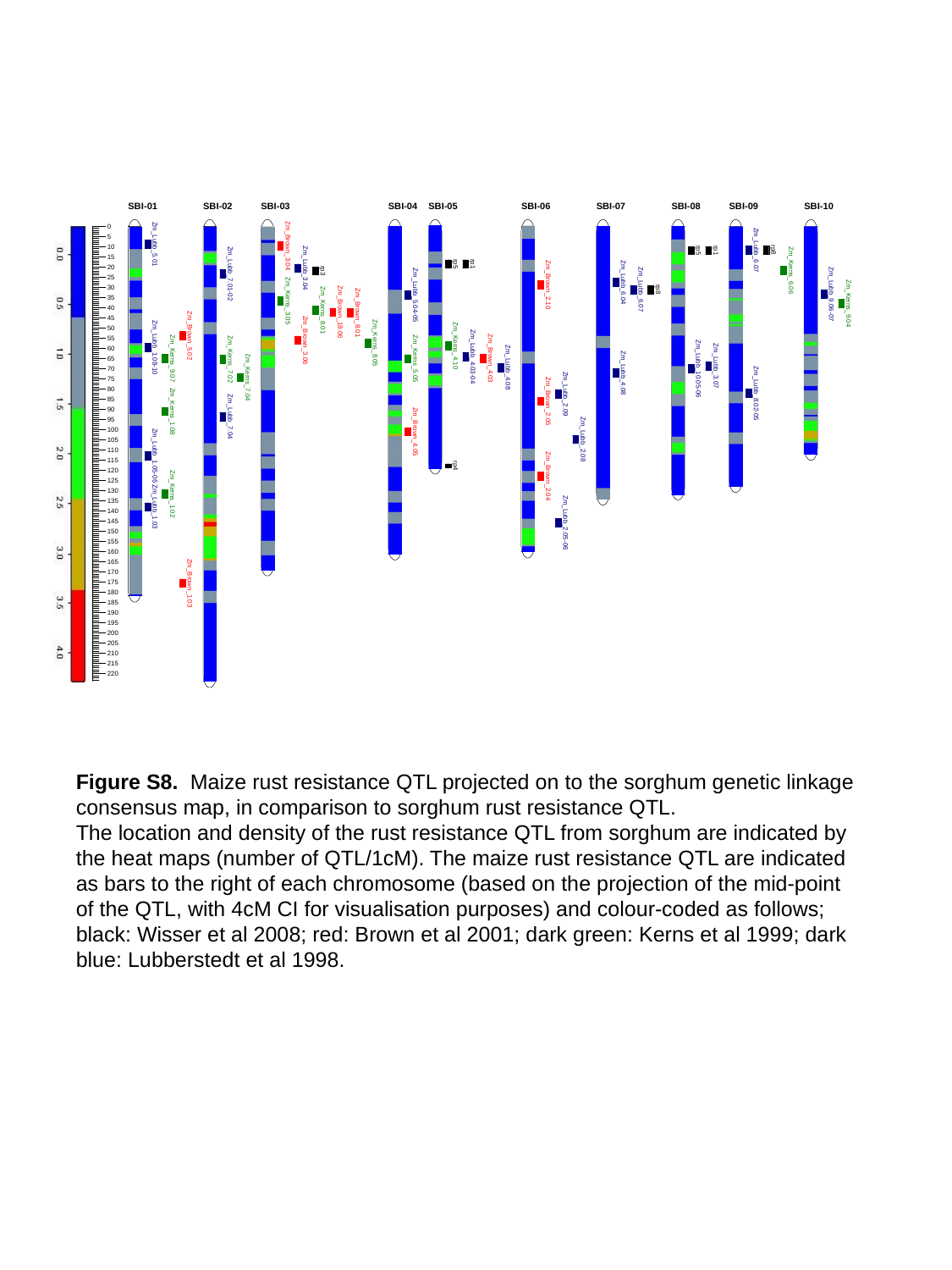

Figure S8. Maize rust resistance QTL projected on to the sorghum genetic linkage consensus map, in comparison to sorghum rust resistance QTL.
The location and density of the rust resistance QTL from sorghum are indicated by the heat maps (number of QTL/1cM). The maize rust resistance QTL are indicated as bars to the right of each chromosome (based on the projection of the mid-point of the QTL, with 4cM CI for visualisation purposes) and colour-coded as follows; black: Wisser et al 2008; red: Brown et al 2001; dark green: Kerns et al 1999; dark blue: Lubberstedt et al 1998.

## Slide 9
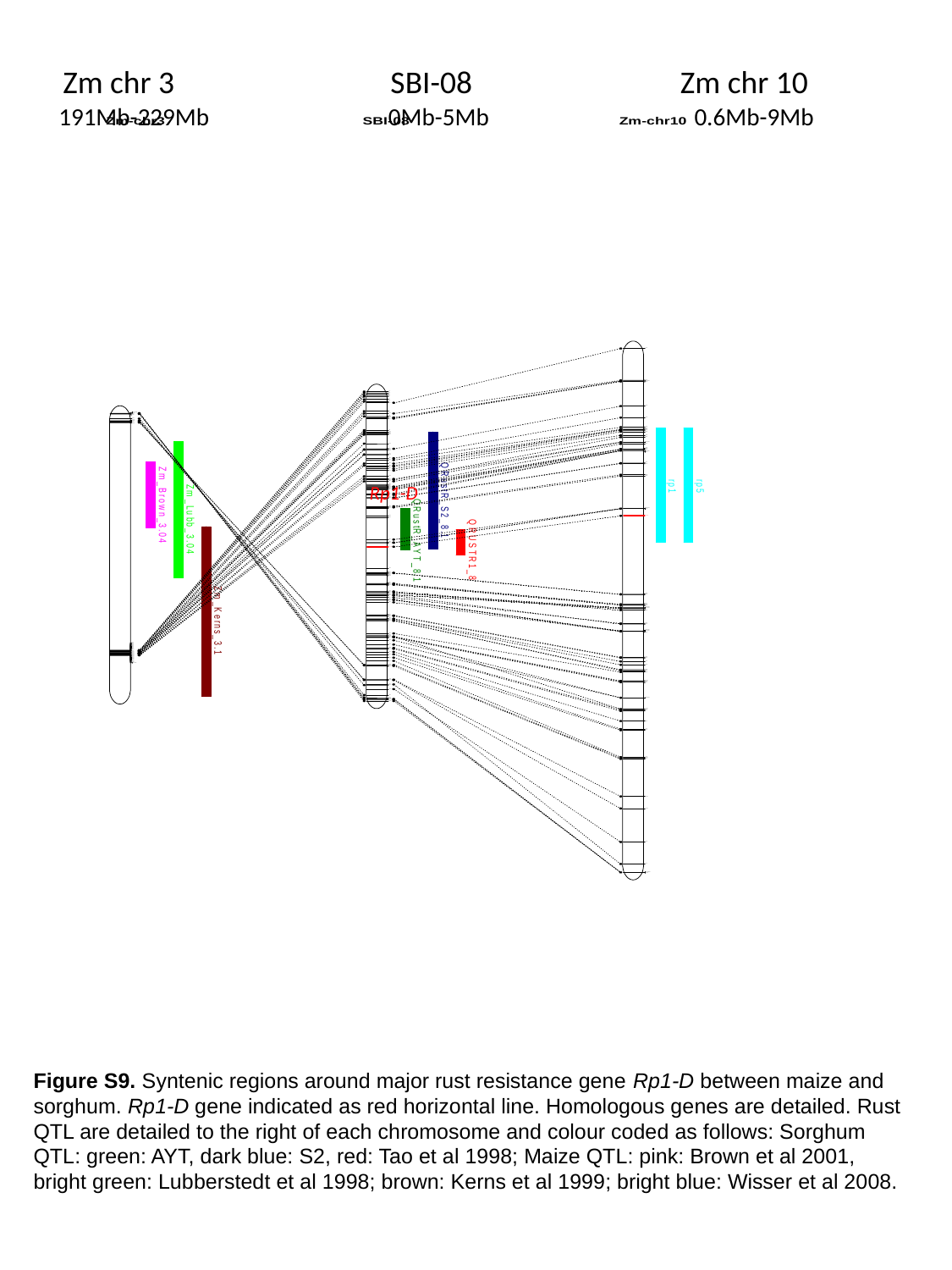

Zm chr 3 SBI-08 Zm chr 10
191Mb-229Mb
0Mb-5Mb
0.6Mb-9Mb
Rp1-D
Figure S9. Syntenic regions around major rust resistance gene Rp1-D between maize and sorghum. Rp1-D gene indicated as red horizontal line. Homologous genes are detailed. Rust QTL are detailed to the right of each chromosome and colour coded as follows: Sorghum QTL: green: AYT, dark blue: S2, red: Tao et al 1998; Maize QTL: pink: Brown et al 2001, bright green: Lubberstedt et al 1998; brown: Kerns et al 1999; bright blue: Wisser et al 2008.

## Slide 10
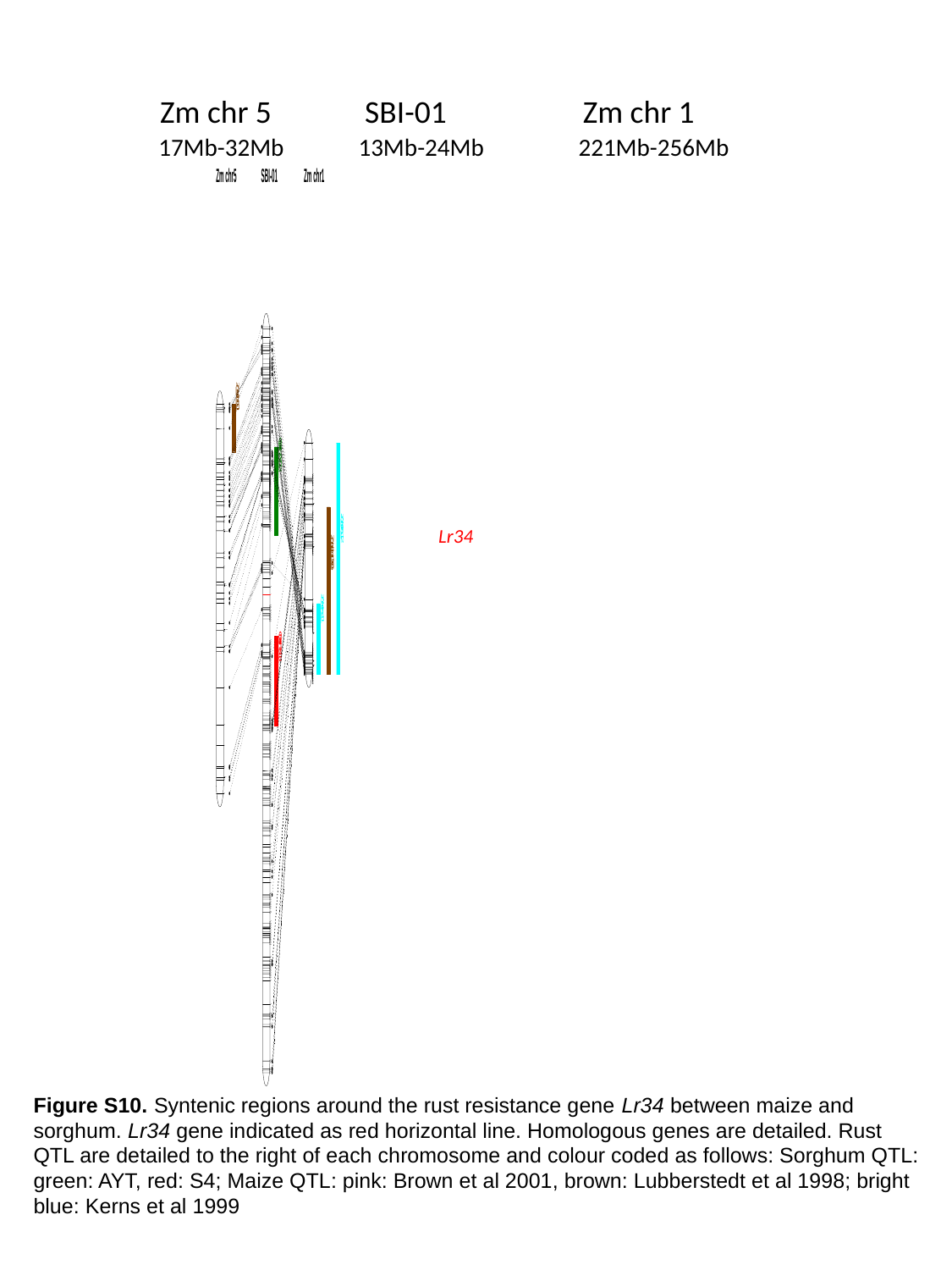

Zm chr 5 SBI-01 Zm chr 1
17Mb-32Mb
13Mb-24Mb
221Mb-256Mb
Lr34
Figure S10. Syntenic regions around the rust resistance gene Lr34 between maize and sorghum. Lr34 gene indicated as red horizontal line. Homologous genes are detailed. Rust QTL are detailed to the right of each chromosome and colour coded as follows: Sorghum QTL: green: AYT, red: S4; Maize QTL: pink: Brown et al 2001, brown: Lubberstedt et al 1998; bright blue: Kerns et al 1999

## Slide 11
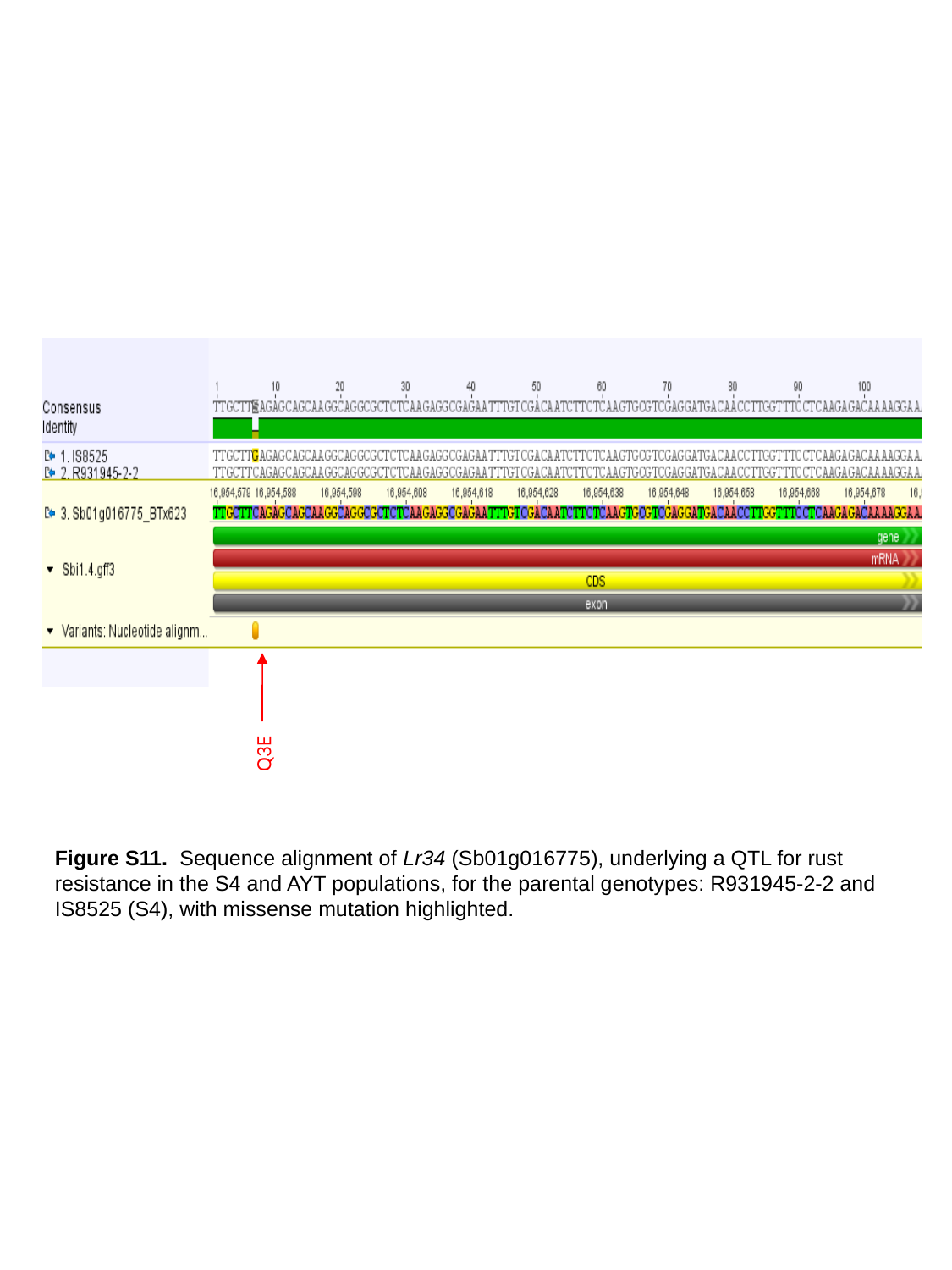

Q3E
Figure S11. Sequence alignment of Lr34 (Sb01g016775), underlying a QTL for rust resistance in the S4 and AYT populations, for the parental genotypes: R931945-2-2 and IS8525 (S4), with missense mutation highlighted.

## Slide 12
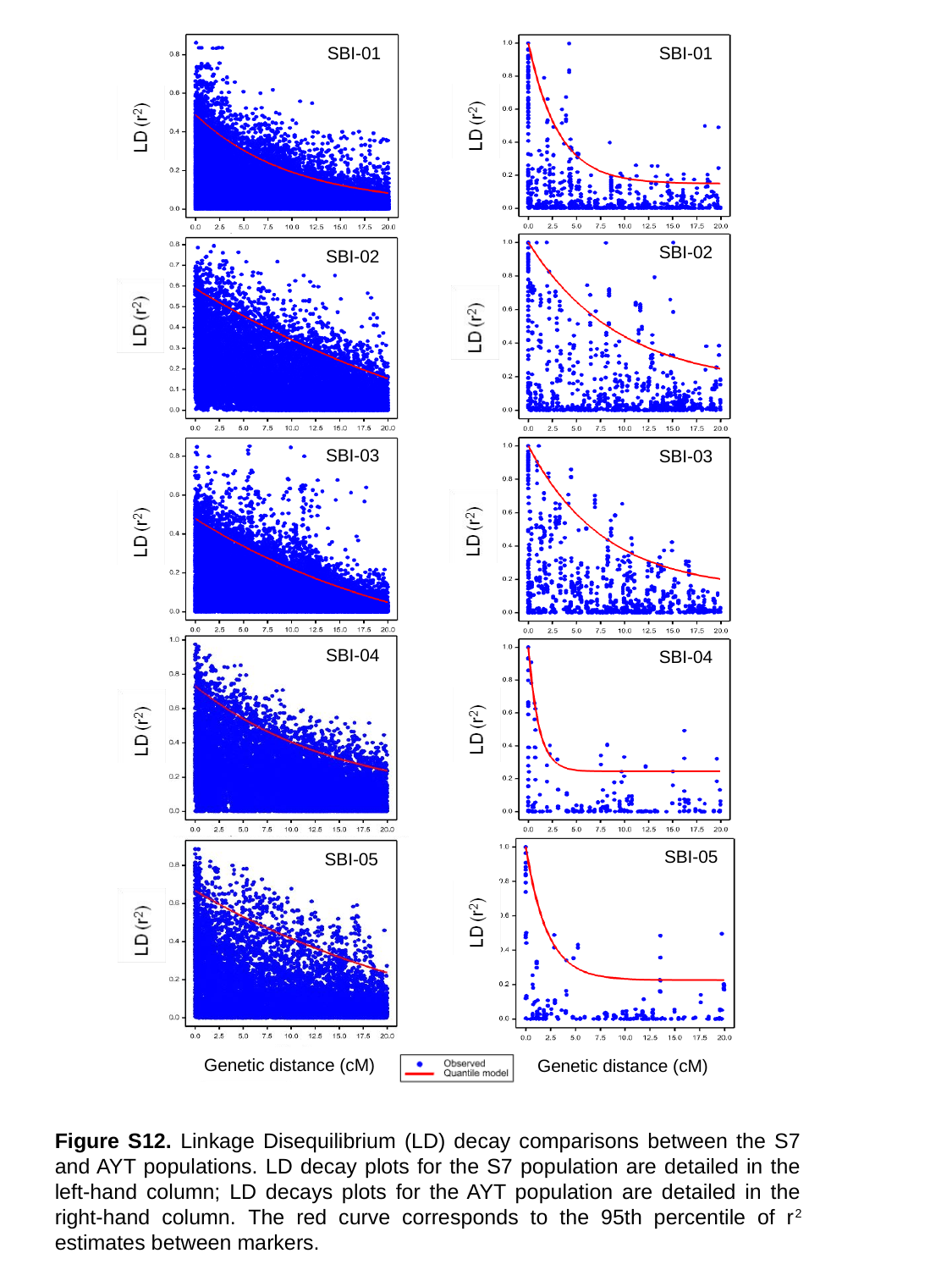

Genetic distance (cM)
Genetic distance (cM)
SBI-01
SBI-01
SBI-02
SBI-02
SBI-03
SBI-03
SBI-04
SBI-04
SBI-05
SBI-05
# Figure S12. Linkage Disequilibrium (LD) decay comparisons between the S7 and AYT populations. LD decay plots for the S7 population are detailed in the left-hand column; LD decays plots for the AYT population are detailed in the right-hand column. The red curve corresponds to the 95th percentile of r2 estimates between markers.

## Slide 13
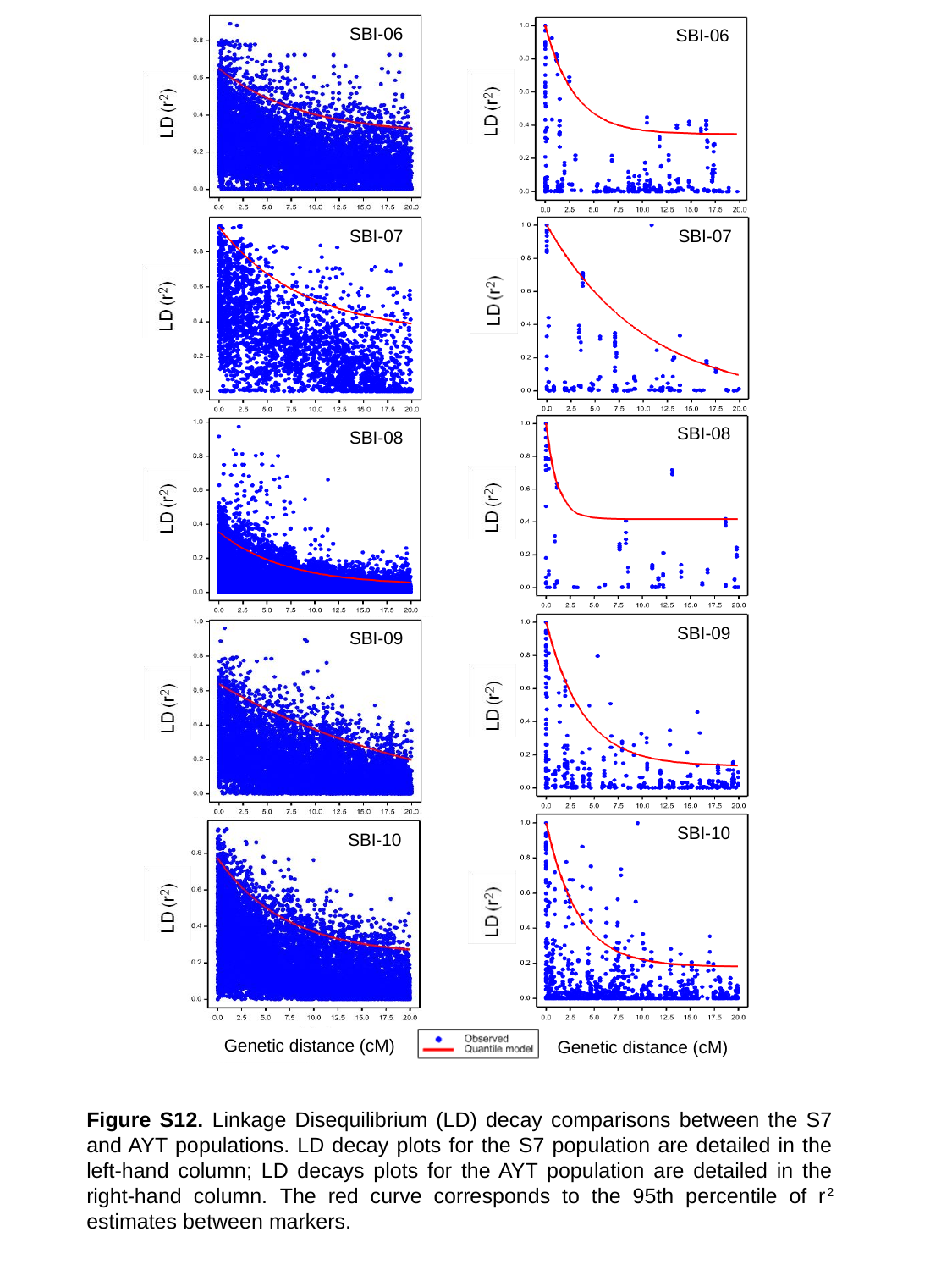

Genetic distance (cM)
Genetic distance (cM)
SBI-06
SBI-06
SBI-07
SBI-07
SBI-08
SBI-08
SBI-09
SBI-09
SBI-10
SBI-10
Figure S12. Linkage Disequilibrium (LD) decay comparisons between the S7 and AYT populations. LD decay plots for the S7 population are detailed in the left-hand column; LD decays plots for the AYT population are detailed in the right-hand column. The red curve corresponds to the 95th percentile of r2 estimates between markers.
