# Supplementary material for: Two distinct classes of QTL determine rust resistance in sorghum
Source: BMC Plant Biol. 2014 Dec 31;14:366. doi: 10.1186/s12870-014-0366-4 (PMC4335369; doi:10.1186/s12870-014-0366-4)
Supplement: Additional file 2: — Contains 8 supplementary tables, all referenced in the main text. [file 12870_2014_366_MOESM2_ESM.doc]

**Two distinct classes of QTL determine rust resistance in sorghum**

**Additional File 1**

**Table S1.** Correlations between traits within 3 bi-parental populations and the AYT association mapping set.

**A**. Correlations of rust score, days to flower (DTF) and height (HGT) in S2 population.

| Trait | rust | DTF | HGT |
| --- | --- | --- | --- |
| rust | 1.00 |  |  |
| DTF | -0.27 | 1.00 |  |
| HGT | -0.08 | 0.07 | 1.00 |

**B**. Correlations of rust score, days to flower (DTF) and height (HGT) in S4 population.

| Trait | rust | DTF | HGT |
| --- | --- | --- | --- |
| Rust | 1.00 |  |  |
| DTF | -0.24 | 1.00 |  |
| HGT | -0.30 | 0.20 | 1.00 |

**C.** Correlations of rust score, days to flower (DTF) and height (HGT) across 2 different sites plus a combined analysis (overall) for S7 population.

| Trait | Site | Rust | | | DTF |  |  | HGT |  |  |
| --- | --- | --- | --- | --- | --- | --- | --- | --- | --- | --- |
|  |  | IRR | nonIRR | overall | IRR | nonIRR | overall | IRR | nonIRR | overall |
| Rust | IRR | 1.00 |  |  |  |  |  |  |  |  |
|  | DRY | 0.94 | 1.00 |  |  |  |  |  |  |  |
|  | overall | 0.99 | 0.98 | 1.00 |  |  |  |  |  |  |
| DTF | IRR | -0.29 | -0.29 | -0.29 | 1.00 |  |  |  |  |  |
|  | DRY | -0.30 | -0.33 | -0.32 | 0.95 | 1.00 |  |  |  |  |
|  | overall | -0.30 | -0.33 | -0.32 | 0.95 | 0.98 | 1.00 |  |  |  |
| HGT | IRR | -0.11 | -0.13 | -0.12 | 0.33 | 0.32 | 0.31 | 1.00 |  |  |
|  | DRY | -0.07 | -0.09 | -0.08 | 0.20 | 0.20 | 0.19 | 0.92 | 1.00 |  |
|  | overall | -0.06 | -0.08 | -0.07 | 0.21 | 0.20 | 0.20 | 0.93 | 0.99 | 1.00 |

**D**. Correlations of rust response between 6 different location/tester combinations in AYT population. Dal986087, Dalby/R986087-2-4-1 combination; Dal993396, Dalby/R993396 combination; Dal995248, Dalby/R995248 combination; Liv986087, Liverpool Plains/R986087-2-4-1 combination; Liv993396, Liverpool Plains/R993396 combination; Liv995248, Liverpool Plains/R995248 combination.

| Location/tester | Dal986087 | Dal993396 | Dal995248 | Liv986087 | Liv993396 | Liv995248 |
| --- | --- | --- | --- | --- | --- | --- |
| Dal986087 | 1.00 |  |  |  |  |  |
| Dal993396 | 0.74 | 1.00 |  |  |  |  |
| Dal995248 | 0.63 | 0.60 | 1.00 |  |  |  |
| Liv986087 | 0.79 | 0.71 | 0.65 | 1.00 |  |  |
| Liv993396 | 0.52 | 0.60 | 0.61 | 0.67 | 1.00 |  |
| Liv995248 | 0.42 | 0.60 | 0.64 | 0.68 | 0.84 | 1.00 |

**Table S2.** Predicted mean ($\bar{X}$) values of rust infection score for the AYT validation set across 6 different location/tester combinations, plus the predicted female standard deviation (σ) and range (minimum and maximum).

| Trait | Location | Tester | X̅ | σ | Min | Max |
| --- | --- | --- | --- | --- | --- | --- |
| Rust | Dalby | R986087-2-4-1 | 3.04 | 0.47 | 2.06 | 5.2 |
|  | Dalby | R993396 | 3.72 | 0.81 | 2.64 | 5.76 |
|  | Dalby | R995248 | 3.18 | 0.72 | 2.14 | 5.2 |
|  | Liverpool Plains | R986087-2-4-1 | 5.53 | 0.85 | 3.47 | 7.44 |
|  | Liverpool Plains | R993396 | 6.65 | 0.91 | 4.91 | 8.24 |
|  | Liverpool Plains | R995248 | 5.79 | 0.82 | 4.24 | 7.91 |
| DTF | Dalby | R986087-2-4-1 | 77.46 | 0.1 | 71.2 | 80.8 |
|  | Dalby | R993396 | 75.16 | 0.09 | 69.53 | 80.52 |
|  | Dalby | R995248 | 74.81 | 0.11 | 70.7 | 80.31 |
|  | Liverpool Plains | R986087-2-4-1 | 79.55 | 0.1 | 72.89 | 83.08 |
|  | Liverpool Plains | R993396 | 76.47 | 0.1 | 70.15 | 81.35 |
|  | Liverpool Plains | R995248 | 78.35 | 0.11 | 74.28 | 83.11 |
| HGT | Liverpool Plains | R986087-2-4-1 | 130.59 | 0.53 | 108.49 | 144.71 |
|  | Liverpool Plains | R993396 | 128.52 | 0.44 | 105.31 | 145.98 |
|  | Liverpool Plains | R995248 | 123.13 | 0.5 | 111.15 | 139.05 |
| LSN | Dalby | R986087-2-4-1 | 2.88 | 0.03 | 2.05 | 4.52 |
|  | Dalby | R993396 | 3.39 | 0.03 | 2.66 | 5.11 |
|  | Dalby | R995248 | 3.38 | 0.03 | 2.64 | 4.28 |
|  | Hermitage | R986087-2-4-1 | 4.47 | 0.06 | 2.87 | 7.57 |
|  | Hermitage | R993396 | 5.49 | 0.06 | 3.43 | 8.63 |
|  | Hermitage | R995248 | 5.82 | 0.07 | 3.99 | 7.71 |

**Table S3.** Mean rust response scores of 4 check varieties included in the five trials detailed in the study.

| Variety | BIL03^IRR^ | BIL03^nonIRR^ | Dalby2011 | Liverpool Plains2011 | HRF2010 |
| --- | --- | --- | --- | --- | --- |
| 86G56 | - | - | 2.02 | 3.94 | 4.00 |
| MR Bonus | 6.67 | 7.13 | - | - | - |
| MR Buster | 6.33 | 7.38 | 2.42 | 6.18 | 6.00 |
| MR Goldrush | 6.00 | 7.20 | - | - | - |

**Table S4.** Summary of rust resistance QTL identified in the AYT association mapping set across 6 different location/tester combinations, detailing the QTL peak position on consensus map, additive effect and significance level.

| QTL | LG | Peak position  (cM) | Allele effects | | | | | |
| --- | --- | --- | --- | --- | --- | --- | --- | --- |
|  |  |  | Dal986087 | Dal993396 | Dal995248 | Liv986087 | Liv993396 | Liv995248 |
| QRustR_AYT_1.1 | SBI-01 | 13.37 | NS | NS | - | 1.135** | NS | - |
| QRustR_AYT_1.2 | SBI-01 | 23.31 | NS | 0.709* | - | 0.912* | NS | NS |
| QRustR_AYT_1.3 | SBI-01 | 37.35 | 0.596* | 0.781** | NS | NS | NS | NS |
| QRustR_AYT_1.4 | SBI-01 | 60 | NS | 0.351* | - | NS | NS | NS |
| QRustR_AYT_1.5 | SBI-01 | 94.88 | 0.522*** | 0.578*** | NS | 0.739*** | 0.663** | NS |
| QRustR_AYT_1.6 | SBI-01 | 109.96 | 0.629** | 0.495** | 0.664** | 0.898** | NS | 0.758* |
| QRustR_AYT_1.7 | SBI-01 | 135.27 | 1.052*** | 0.996*** | 0.633* | 1.050*** | NS | NS |
| QRustR_AYT_1.8 | SBI-01 | 157.57 | 0.583** | 0.571** | 0.660** | NS | NS | NS |
| QRustR_AYT_2.1 | SBI-02 | 15.38 | 0.326** | 0.316** | 0.407*** | 0.500*** | 0.459*** | 0.556*** |
| QRustR_AYT_2.2 | SBI-02 | 32.2 | NS | 0.262** | NS | 0.339* | NS | NS |
| QRustR_AYT_2.3 | SBI-02 | 49.3 | 0.202* | NS | NS | NS | NS | NS |
| QRustR_AYT_2.4 | SBI-02 | 109.33 | 1.043*** | 0.987*** | 1.051* | 1.046*** | NS | NS |
| QRustR_AYT_2.5 | SBI-02 | 123.5 | 1.1** | 1.011* | NS | 1.052*** | 0.826*** | 0.886*** |
| QRustR_AYT_2.6 | SBI-02 | 144.31 | 0.710** | 0.666** | 1.059** | 0.815** | NS | 0.815*** |
| QRustR_AYT_2.7 | SBI-02 | 166.77 | 0.633** | 0.649** | NS | 0.774** | 0.534* | NS |
| QRustR_AYT_2.8 | SBI-02 | 182.56 | NS | 0.528* | NS | NS | NS | NS |
| QRustR_AYT_3.1 | SBI-03 | 2.79 | 0.654*** | 0.561*** | 0.667*** | 0.791*** | NS | 0.709** |
| QRustR_AYT_3.2 | SBI-03 | 29.51 | NS | 0.526* | NS | NS | NS | NS |
| QRustR_AYT_3.3 | SBI-03 | 46.69 | NS | NS | 0.360** | 1.118** | NS | NS |
| QRustR_AYT_3.4 | SBI-03 | 57.78 | 0.212* | 0.169* | NS | 0.301* | NS | NS |
| QRustR_AYT_3.5 | SBI-03 | 64.45 | NS | NS | 0.175** | 0.402* | NS | 0.260* |
| QRustR_AYT_3.6 | SBI-03 | 116.64 | NS | 0.171* | NS | NS | NS | NS |
| QRustR_AYT_3.7 | SBI-03 | 156.18 | 1.068* | 0.991** | 0.783* | 1.005** | NS | NS |
| QRustR_AYT_4.1 | SBI-04 | 71.45 | NS | NS | 0.371* | 0.538*** | 0.408* | 0.683*** |
| QRustR_AYT_4.2 | SBI-04 | 83.54 | NS | NS | NS | NS | 0.299** | 0.376** |
| QRustR_AYT_4.3 | SBI-04 | 94.4 | NS | 0.262** | NS | 0.503** | 0.343** | 0.395* |
| QRustR_AYT_4.4 | SBI-04 | 102.66 | 1.070* | 0.982* | 0.480* | NS | NS | NS |
| QRustR_AYT_4.5 | SBI-04 | 138.71 | NS | NS | 0.382** | NS | NS | NS |
| QRustR_AYT_4.6 | SBI-04 | 149.18 | NS | NS | NS | 0.436* | NS | NS |
| QRustR_AYT_5.1 | SBI-05 | 23.21 | 0.851*** | 0.522** | - | 1.021*** | NS | 0.940** |
| QRustR_AYT_5.2 | SBI-05 | 40.77 | 0.253* | NS | - | 0.449*** | NS | NS |
| QRustR_AYT_5.3 | SBI-05 | 56.8 | NS | - | - | 1.127** | - | - |
| QRustR_AYT_5.4 | SBI-05 | 64.31 | 0.513** | 0.452* | NS | 0.730*** | NS | NS |
| QRustR_AYT_5.5 | SBI-05 | 76.07 | NS | NS | NS | NS | NS | 0.303** |
| QRustR_AYT_6.1 | SBI-06 | 2.74 | 0.394* | NS | NS | NS | NS | NS |
| QRustR_AYT_6.2 | SBI-06 | 65.94 | NS | NS | NS | NS | NS | 0.269* |
| QRustR_AYT_6.3 | SBI-06 | 112.82 | 0.538*** | 0.448* | 0.603*** | 0.711** | NS | 0.579* |
| QRustR_AYT_6.4 | SBI-06 | 125.35 | 0.741* | 0.763* | 0.670** | NS | NS | NS |
| QRustR_AYT_6.5 | SBI-06 | 147.78 | 0.532*** | 0.410** | 0.579** | 0.729*** | 0.393* | 0.690** |
| QRustR_AYT_7.1 | SBI-07 | 54.52 | NS | NS | NS | 1.114** | NS | 0.465* |
| QRustR_AYT_8.1 | SBI-08 | 15.57 | 0.63* | 0.644** | NS | 0.771** | NS | NS |
| QRustR_AYT_8.2 | SBI-08 | 36.75 | 0.883*** | 0.850*** | 0.879*** | 0.884*** | NS | NS |
| QRustR_AYT_8.3 | SBI-08 | 50.76 | NS | NS | 0.258* | NS | NS | NS |
| QRustR_AYT_8.4 | SBI-08 | 79.6 | 0.746*** | 0.725*** | NS | 0.774** | NS | NS |
| QRustR_AYT_9.1 | SBI-09 | 24.06 | NS | NS | 0.716** | NS | NS | 0.835** |
| QRustR_AYT_9.2 | SBI-09 | 34.33 | 0.317* | NS | 0.413** | NS | NS | NS |
| QRustR_AYT_9.3 | SBI-09 | 47.2 | 0.422* | NS | 0.561** | NS | NS | NS |
| QRustR_AYT_10.1 | SBI-10 | 54.3 | 0.653** | 0.700** | 0.361*** | 0.900* | NS | NS |
| QRustR_AYT_10.2 | SBI-10 | 64.52 | 1.082** | 0.994*** | 1.068** | 1.011*** | 0.773** | 0.826** |
| QRustR_AYT_10.3 | SBI-10 | 81.39 | 1.050*** | 0.994*** | 0.467** | 1.048*** | NS | NS |
| QRustR_AYT_10.4 | SBI-10 | 96.74 | 0.669** | NS | 0.775*** | 0.884** | NS | 0.892*** |
| QRustR_AYT_10.5 | SBI-10 | 102.89 | NS | NS | 0.597*** | NS | NS | 0.646** |

* indicates suggestive (–log10P ≥ 3.5); **indicates significant ( –log10P ≥ 4); ***indicates highly significant ( –log10P ≥ 5); NS indicates not significant.

**Table S5.** Summary of QTL for days to flower (DTF) identified in the S7 population across 2 trials in 2003, detailing the QTL location on consensus map, additive effect and significance level. IRR, irrigated conditions; nonIRR, non-irrigated conditions; combined analysis, combined analysis across two trials.

| QTL ID | LG | Support interval (cM) | Position (cM)^a^ | IRR | nonIRR | Combined analysis |
| --- | --- | --- | --- | --- | --- | --- |
| QDTF_S7_1.1 | SBI-01 | 65.1-70.1 | 70.04 | NS | -0.269*** | NS |
| QDTF_S7_1.2 | SBI-01 | 171.7-182.9 | 181.7 | 0.518*** | 0.431*** | 0.631** |
| QDTF_S7_2.1 | SBI-02 | 55.6-93.7 | 62.2 | -0.351** | -0.329*** | -0.488* |
| QDTF_S7_3.1 | SBI-03 | 21.3-22.1 | 21.3 | 0.321** | 0.283*** | 0.453** |
| QDTF_S7_3.2 | SBI-03 | 144.5 | 144.5 | 0.303** | 0.266*** | 0.422** |
| QDTF_S7_6.1 | SBI-06 | 22.9 | 22.9 | 0.608*** | 0.459*** | NS |
| QDTF_S7_6.2 | SBI-06 | 125.6-165.1 | 162.1 | 0.605*** | 0.483*** | 0.725*** |
| QDTF_S7_7.1 | SBI-07 | 0-24.9 | 1.45 | 0.493*** | 0.333*** | 0.469** |
| QDTF_S7_8.1 | SBI-08 | 4.1-11.9 | 5.05 | 0.623*** | 0.498*** | 0.664** |
| QDTF_S7_8.2 | SBI-08 | 24.5-30.3 | 24.54 | 0.842*** | 0.586*** | 1.056*** |
| QDTF_S7_8.3 | SBI-08 | 48.2-52.4 | 48.25 | 0.799*** | 0.578*** | 1.055*** |
| QDTF_S7_8.4 | SBI-08 | 73.2-96.7 | 73.25 | 0.645*** | 0.438*** | 0.876*** |
| QDTF_S7_9.1 | SBI-09 | 5.1-5.6 | 5.13 | 0.343** | 0.284** | NS |
| QDTF_S7_9.2 | SBI-09 | 63.8-90.6 | 87.2 | 0.636*** | 0.492*** | 0.926*** |
| QDTF_S7_10.1 | SBI-10 | 31.8-38.7 | 38.01 | 0.505*** | 0.379*** | 0.550** |
| QDTF_S7_10.2 | SBI-10 | 48.9 | 48.89 | 0.643*** | 0.478** | NS |
| QDTF_S7_10.3 | SBI-10 | 57.9 | 57.85 | 0.654*** | 0.453*** | 0.689** |
| QDTF_S7_10.4 | SBI-10 | 76.3 | 76.3 | 0.358** | 0.289** | 0.492** |

^a^Peak position in cM with maximum –log_10_P; NS indicates not significant; ** indicates significant (-log10P ≥ 3); *** indicates highly significant (-log10P ≥ 4).

**Table S6.** Summary of QTL for maturity and height identified in the AYT association mapping set across 6 different location/tester combinations, detailing the QTL peak position on consensus map, additive effect and significance level.

| QTL | LG | Peak position  (cM) | Allele effects | | | | | |
| --- | --- | --- | --- | --- | --- | --- | --- | --- |
|  |  |  | Dal986087 | Dal993396 | Dal995248 | Liv986087 | Liv993396 | Liv995248 |
| QDTF_AYT_1.1 | SBI-01 | 171.82 | -2.128*** | -2.261*** | -1.624* | -2.201*** | -2.185** | -1.671* |
| QDTF_AYT_2.1 | SBI-02 | 147.67 | -1.983*** | -2.099*** | NS | -1.786** | -2.032*** | NS |
| QDTF_AYT_2.2 | SBI-02 | 166.77 | -1.608* | NS | -2.16*** | NS | NS | -2.116** |
| QDTF_AYT_3.1 | SBI-03 | 156.64 | NS | -2.071** | -1.858** | NS | NS | -1.86* |
| QDTF_AYT_4.1 | SBI-04 | 139.73 | NS | NS | 1.205** | NS | NS | NS |
| QDTF_AYT_8.1 | SBI-08 | 15.57 | -1.599* | NS | -2.18*** | NS | NS | -2.121** |
| QDTF_AYT_9.1 | SBI-09 | 24.06 | -1.636*** | -1.632*** | NS | -1.782*** | -1.596*** | NS |
| QDTF_AYT_9.2 | SBI-09 | 47.2 | NS | NS | NS | NS | NS | 0.846* |
| QDTF_AYT_9.3 | SBI-09 | 64.91 | NS | NS | NS | 0.74* | NS | NS |
| QDTF_AYT_10.1 | SBI-10 | 64.52 | NS | -1.528*** | NS | NS | -1.306** | NS |
| QHGT_AYT_9.1 | SBI-09 | 47.20 | - | - | - | NS | NS | 6.29* |

* indicates significant at –log10P ≥ 3.5; **indicates significant at –log10P ≥ 4; ***indicates highly significant ( –log10P ≥ 5); NS indicates not significant; - indicates not available.

**Table S7.** Summary of QTL for height (HGT) identified in the S7 population across 2 trials in 2003, detailing the QTL location on consensus map, additive effect and significance level. IRR, irrigated conditions; nonIRR, non-irrigated conditions; combined analysis, combined analysis across two trials.

| QTL ID | LG | Support interval (cM) | Position (cM)^a^ | IRR | nonIRR | Combined analysis |
| --- | --- | --- | --- | --- | --- | --- |
| QHGT_S7_3.1 | SBI-03 | 117.8 | 117.8 | NS | 3.531** | 2.637** |
| QHGT_S7_4.1 | SBI-04 | 56.4-56.6 | 56.46 | NS | 3.589** | 2.803** |
| QHGT_S7_6.1 | SBI-06 | 84.1 | 84.18 | 2.863** | 3.015** | 2.308** |
| QHGT_S7_6.2 | SBI-06 | 115.9 | 115.93 | 3.036** | NS | 2.207** |
| QHGT_S7_9.1 | SBI-09 | 127.6-128.5 | 127.95 | 4.217*** | 5.364*** | 3.957*** |
| QHGT_S7_10.1 | SBI-10 | 75.1 | 75.11 | NS | NS | -2.405** |

^a^Peak position in cM with maximum –log_10_P; NS indicates not significant; ** indicates significant (-log10P ≥ 3); *** indicates highly significant (-log10P ≥ 4).

**Table S8.** Summary of QTL for LSN (leaf senescence) identified in the AYT association mapping set across 6 different location/tester combinations, detailing the QTL peak position on consensus map, additive effect and significance level.

| QTL | LG | Peak position  (cM) | Allele effects | | | | | |
| --- | --- | --- | --- | --- | --- | --- | --- | --- |
|  |  |  | Dal986087 | Dal993396 | Dal995248 | Herm986087 | Herm993396 | Herm995248 |
| QLSN_AYT_1.1 | SBI-01 | 115.21 | 0.331* | NS | NS | NS | NS | NS |
| QLSN_AYT_1.2 | SBI-01 | 138.99 | NS | 0.533** | 0.179* | NS | 1.305* | - |
| QLSN_AYT_2.1 | SBI-02 | 109.33 | NS | 0.682** | NS | NS | 1.296* | NS |
| QLSN_AYT_3.1 | SBI-03 | 2.79 | 0.402* | NS | NS | 0.881** | NS | NS |
| QLSN_AYT_3.2 | SBI-03 | 39.07 | - | 0.291* | - | - | - | - |
| QLSN_AYT_8.1 | SBI-08 | 36.75 | NS | 0.511* | NS | NS | 1.113* | NS |
| QLSN_AYT_8.2 | SBI-08 | 79.6 | 0.442* | NS | NS | NS | NS | NS |
| QLSN_AYT_10.1 | SBI-10 | 34.22 | 0.411** | 0.423** | NS | NS | NS | NS |
| QLSN_AYT_10.2 | SBI-10 | 88.89 | NS | 0.679** | - | NS | 1.296* | - |

* indicates significant at –log10P ≥ 3.5; **indicates significant at –log10P ≥ 4; NS indicates not significant; - indicates not available.
